# A personalized classification of behavioral severity of autism spectrum disorder using a comprehensive machine learning framework

**DOI:** 10.1038/s41598-023-43478-z

**Published:** 2023-10-09

**Authors:** Mohamed T. Ali, Ahmad Gebreil, Yaser ElNakieb, Ahmed Elnakib, Ahmed Shalaby, Ali Mahmoud, Ahmed Sleman, Guruprasad A. Giridharan, Gregory Barnes, Ayman S. Elbaz

**Affiliations:** 1 Bioengineering Department, University of Louisville, Louisville, KY 40292 USA; 2 UT Southwestern Medical Center, UT Southwestern Medical Center, Dallas, TX 75390 USA; 3 Electrical and Computer Engineering, Penn State Erie-The Behrend College, Erie, PA 16563 USA; 4 Lyda Hill Department of Bioinformatics, UT Southwestern Medical Center, Dallas, TX 75390 USA; 5 Department of Neurology and Pediatric Research Institute, University of Louisville, Louisville, KY 40202 USA

**Keywords:** Engineering, Biomedical engineering, Neuroscience, Computational neuroscience, Neural circuits, Neurology, Neurological disorders, Neurodevelopmental disorders

## Abstract

Autism Spectrum Disorder (ASD) is characterized as a neurodevelopmental disorder with a heterogeneous nature, influenced by genetics and exhibiting diverse clinical presentations. In this study, we dissect Autism Spectrum Disorder (ASD) into its behavioral components, mirroring the diagnostic process used in clinical settings. Morphological features are extracted from magnetic resonance imaging (MRI) scans, found in the publicly available dataset ABIDE II, identifying the most discriminative features that differentiate ASD within various behavioral domains. Then, each subject is categorized as having severe, moderate, or mild ASD, or typical neurodevelopment (TD), based on the behavioral domains of the Social Responsiveness Scale (SRS). Through this study, multiple artificial intelligence (AI) models are utilized for feature selection and classifying each ASD severity and behavioural group. A multivariate feature selection algorithm, investigating four different classifiers with linear and non-linear hypotheses, is applied iteratively while shuffling the training-validation subjects to find the set of cortical regions with statistically significant association with ASD. A set of six classifiers are optimized and trained on the selected set of features using 5-fold cross-validation for the purpose of severity classification for each behavioural group. Our AI-based model achieved an average accuracy of 96%, computed as the mean accuracy across the top-performing AI models for feature selection and severity classification across the different behavioral groups. The proposed AI model has the ability to accurately differentiate between the functionalities of specific brain regions, such as the left and right caudal middle frontal regions. We propose an AI-based model that dissects ASD into behavioral components. For each behavioral component, the AI-based model is capable of identifying the brain regions which are associated with ASD as well as utilizing those regions for diagnosis. The proposed system can increase the speed and accuracy of the diagnostic process and result in improved outcomes for individuals with ASD, highlighting the potential of AI in this area.

## Introduction

According to the Diagnostic and Statistical Manual of Mental Disorders (DSM-5), autism spectrum disorders (ASD) are neurodevelopmental disorders with common impairments in social communication and interactions, and restricted and repetitive behavioral patterns^1^. ASD is also described as a heterogeneous neurodevelopmental disorder that has a strong genetic basis and various clinical presentations^2^. The subtypes of ASD vary in terms of the severity of symptoms, associated language and cognitive abilities, and the patterns of symptoms^3^. The Centers for Disease Control and Prevention in the US has reported^4^ that, for the last few years, ASD prevalence has been increasing, especially among children, reaching almost one in 58. ASD individuals and their families face a substantial financial and emotional burden, as the incidence of ASD continues to rise. Additionally, ASD places increased pressure on the medical, social, and political aspects of society^5^.

ASD is considered a “spectrum disorder” because of the wide variations in the type and severity of symptoms that people with ASD experience^6^. The latest edition of DSM-5 categorizes ASD as encompassing spectrum disorder and discards the previous DSM-IV, which denotes variations of the spectrum: autistic disorder, Asperger syndrome, childhood disintegrative disorder, and pervasive developmental disorders not otherwise specified (PDD-NOS)^7^. As mentioned earlier, patients on the spectrum exhibit a variety of behavioral anomalies; each of those behavioral traits can be thought of as an independent dimension describing ASD. Thus, we can conceptualize ASD as a multidimensional disorder with each dimension referring to a specific symptom (or anomalous behavioral trait) with a given severity. The conceptualization of ASD as a heterogeneous multi-dimensional disorder had been addressed in previous studies^8–11^.

We incorporated this concept into a comprehensive machine-learning framework to achieve a better understanding of the disorder as well as higher classification performance.

The gold standard for diagnosing ASD is a combination of the Autism Diagnostic Observation Schedule (ADOS) and Autism Diagnostic Interview-Revised (ADI-R)^12^. ADOS and ADI-R diagnose autism through an interviewing procedure with the subject. Throughout the interview process, the physician scores the subject based on several behavioral traits including social reciprocity, verbal or nonverbal communication, and repetitive behavior^12,13^, thus we can think of the diagnosis process of the gold standard as measuring the severity of autism in each dimension. Despite being the gold standard, ADOS and ADI-R require extensive training and are very time-consuming^14^. Furthermore, the symptoms of autism are very wide-ranging to the point that raises suspicions of autism in the individual^15,16^, as well as the physician’s experience, the behavior of the patient, and the knowledge of the parent make the diagnostic process highly subjective^1,15^. Given the limitations of the gold standard, an *in vivo* tool is urgently required to diagnose, or asses the severity of, ASD. An alternative diagnostic instrument for ASD is the Social Responsiveness Scale (SRS), which is quickly accomplished, objective, economical, easy to use, and increasingly being used as a clinical screening tool^17,18^. In the work presented here, we utilized the standardized SRS score as the ground truth for the severity of each behavioral trait. The main rationale behind selecting SRS instead of ADOS or ADI-R is that the typically developed (TD) subjects in the dataset utilized in this study (Autism Brain Imaging Data Exchange II) do not possess any ADOS or ADI-R scores. However, the methodology proposed in this study is applicable to any behavioral scoring system including, and not limited to, SRS, ADOS and ADI-R.

In this study, we classify ASD by situating patients in a multi-dimensional behavioral space. A multi-dimensional behavioral space is defined by an array of SRS behavioral scores for each subject. Thus, we identify the location of each subject on the spectrum via identifying the severity of his or her symptoms along each behavioral axis. Furthermore, we identify the cortical regions which are most correlated with the severity of each behavioral category. To the best of our knowledge, we are the first team to classify ASD subjects based on their predicted behavioral severity scores, and annotate the most correlated brain region with each behavioral category. The prior work we believe closest to our own is the study conducted by Moradi et al.,^19^ who predicted the symptoms severity score of individuals with ASD based solely on cortical thickness. In the following section, we will cover Moradi et al. study as well as the most recent studies that classify ASD using machine learning and structural MRI (sMRI).

### Related work

Moradi and colleagues^19^ attempted to use support vector regression (SVR) and Elastic Net penalized linear regression to predict the severity of symptoms in individuals with ASD based exclusively on cortical thickness. The authors included 156 individuals with ASD. Their subjects were compiled from four sites in the Autism Brain Imaging Data Exchange 1 (ABIDE-1). ASD severity score was based on the participants’ ADOS. The researchers observed an average mean absolute error of 1.36 and an average correlation of 0.51. Although the work of Moradi et al.^19^ is significant, it lacks a sufficient number of subjects to generalize the results, as well as utilizes only one morphological feature. Dekhil et al.^20^ utilized fMRI and sMRI modalities to extract features from 185 subjects. Those data were downloaded from the National Database for Autism Research (NDAR). The authors reported that using fMRI data alone, they achieved 75% classification accuracy, using sMRI data alone they achieved 79% classification accuracy, and when they fused both features together, they achieved 81% accuracy. The same limitation of Moradi et al.^19^ applied to Dekhil et al.^20^, where the number of subjects is too few for generalization. Yassin et al.^21^ employed various machine learning (ML) methods to perform multi-class classification between TD, ASD, and schizophrenia subjects, as well as binary classification between each pair of classes. The study included 36 ASD, 106 TD, and 64 schizophrenia subjects. The authors reported classification accuracies of 69% for multi-class classification, 75% for ASD vs. schizophrenia classification, 75.8% for ASD vs. TD classification, and 70.6% for schizophrenia vs. TD classification. The Yassin et al. study shares the same limitations as Dekhil et al. and Moradi et al. Ali et. al.^22^ proposed a feature selection algorithm to select the most relevant morphological features to ASD that maximize the classification accuracy. The authors utilized ABIDE I dataset, and morphological features extracted T1-weighted sMRI to classify ASD. The authors achieved an accuracy of 82% using neural networks, and 72% using support vector machines. Although the Ali et al. study solves the limitation of using few subjects, the study doesn’t provide any insights regarding the behavioral reports.

In this study, we propose a novel personalized comprehensive ML model to dissect ASD into behavioral components according to the SRS. We classify the severity of each behavioral category as they are defined in the SRS modules (Communication, Mannerism, Cognition, Motivation, Awareness, and Total). The severity levels are defined as follows: (1) TD and (2) mild, (3) moderate, and (4) severe, such that mild, moderate, and severe are all considered to be ASD. We search for the neuroimaging markers that define every severity level (TD, mild, moderate, and severe) of each behavioral category. Those neuroimaging markers are used for training a machine learning model to classify subjects based on their severity level (TD, mild, moderate, or severe) in each behavioral category. This experiment is repeated 51 times while shuffling the training validation set. The cortical regions with statistically significant classification accuracy are selected for each behavioral module to build the corresponding behavioral neuro-atlas. The primary reasons for relying exclusively on morphological features extracted from the brain cortex and disregarding the subcortical structures are as follows: (1) segmentation of subcortical structures is more difficult and more susceptible to errors compared to cortex segmentation and (2) most significant discoveries in previous studies are based on surface-based morphometry (SBM) techniques^2,15,21,23–30^. The primary motivations behind utilizing the SRS instead of other instruments as our ground truth are: (1) It is cost-efficient and less time-consuming than the gold standard. (2) More complete data are available in the source database for SRS compared to other metrics; in particular, TD subjects have no ADOS data in ABIDE II.

In this study, we avoided the limitations that existed in the aforementioned literature such as: (1) working on a small sample size^21,30^, and (2) neglecting the heterogeneity nature of ASD^20–22,31^. To overcome these limitations, we propose the following features: (1) we utilized ABIDE II dataset comprises 521 individuals with ASD and 593 TD subjects^32^ and (2) we split ASD into its behavioral components as described by SRS, and within each behavioral component, we classify subjects according to their severity to mitigate each of those limitations, respectively. To the best of our knowledge, we are the first team to build a Computer Aided Diagnosis (CAD) system that simulates the clinic environment by diagnosing ASD in terms of the behavioral scores of SRS.

**Figure 1:**
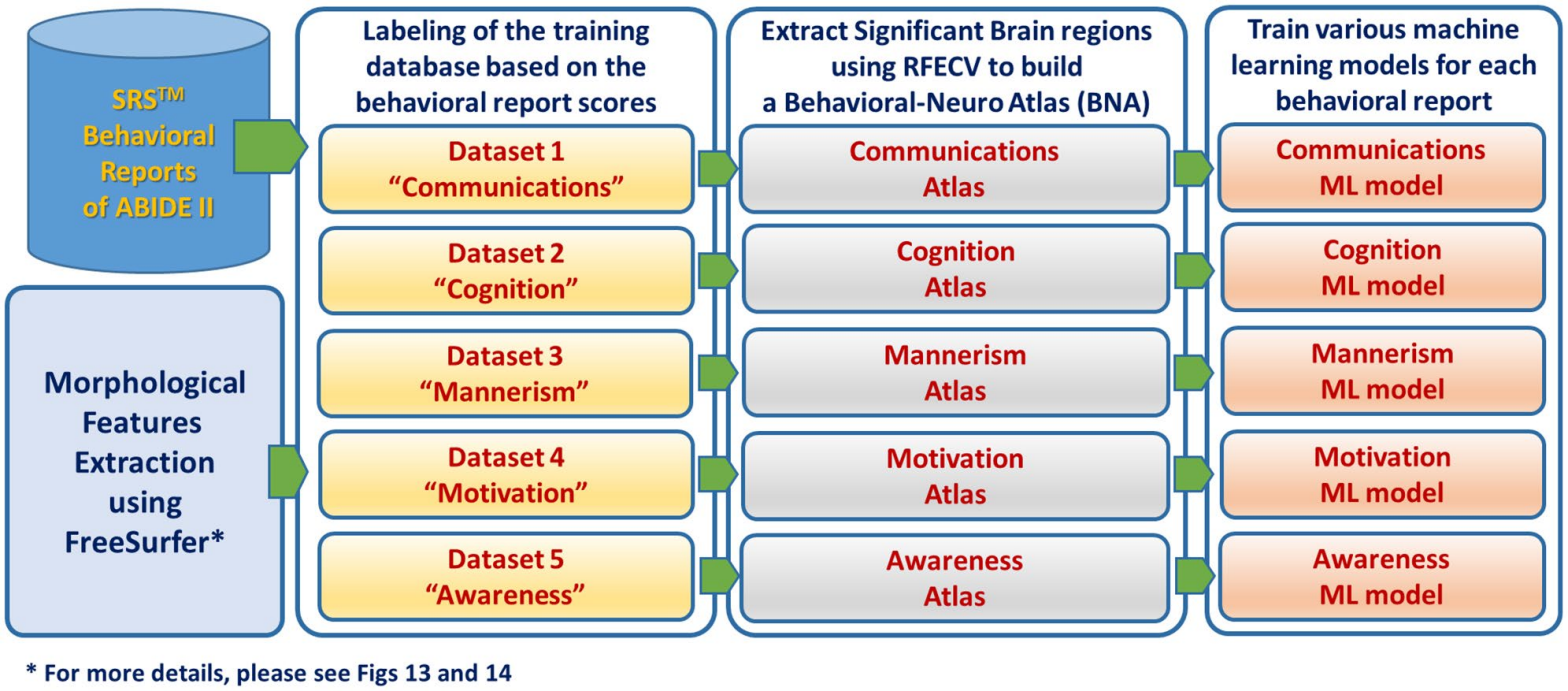
An outline of the proposed approach, starting from the acquisition of MRI volumes to the diagnosis. Note that the training of the ML models in the right panel involves hyperparameter optimization.

We hypothesize that the optimum classification results of autism can be achieved by splitting ASD into behavioral components and predicting each behavioral component. The main contributions of the proposed approach (Fig. 1) can be summarized as follows: (1) modeling ASD as a multidimensional disorder such that each behavioral component is an independent dimension, and the severity of ASD is the magnitude of ASD in that dimension, (2) building a comprehensive ML pipeline to find morphological features and brain regions that are correlated with different severity levels of each behavioral category of the SRS, (3) finding the anomalous neuro-circuits caused by different severity levels of autism (e.g. neuro-atlases), and (4) proposing an explanatory CAD report which maps extracted cortical features to the behavioral classification to the final diagnosis.

## Results

In this section, we will cover the output of the behavioral classification step. We focus on justifying the results of neuro-atlases, as well as, we are hoping that those neuro-atlases would help more scientists to understand ASD etiology.

### Neuro-atlases

**Figure 2:**
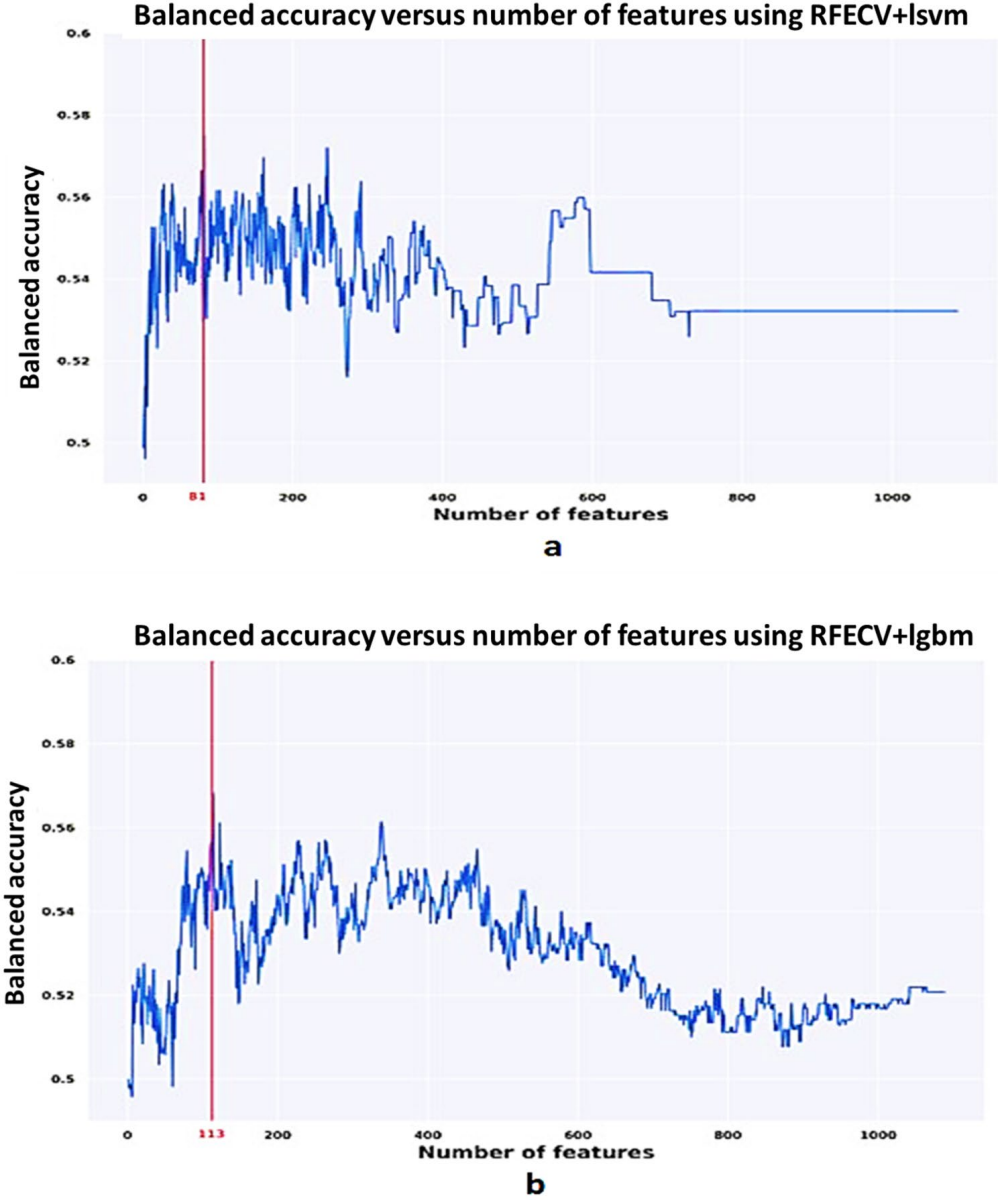
Balanced accuracy versus the number of selected features using each of Recursive Feature Elimination with Cross-Validation (RFECV) classifiers. (**a**) Linear support vector machines, (**b**) Light Gradient Boosting machines.

**Figure 3:**
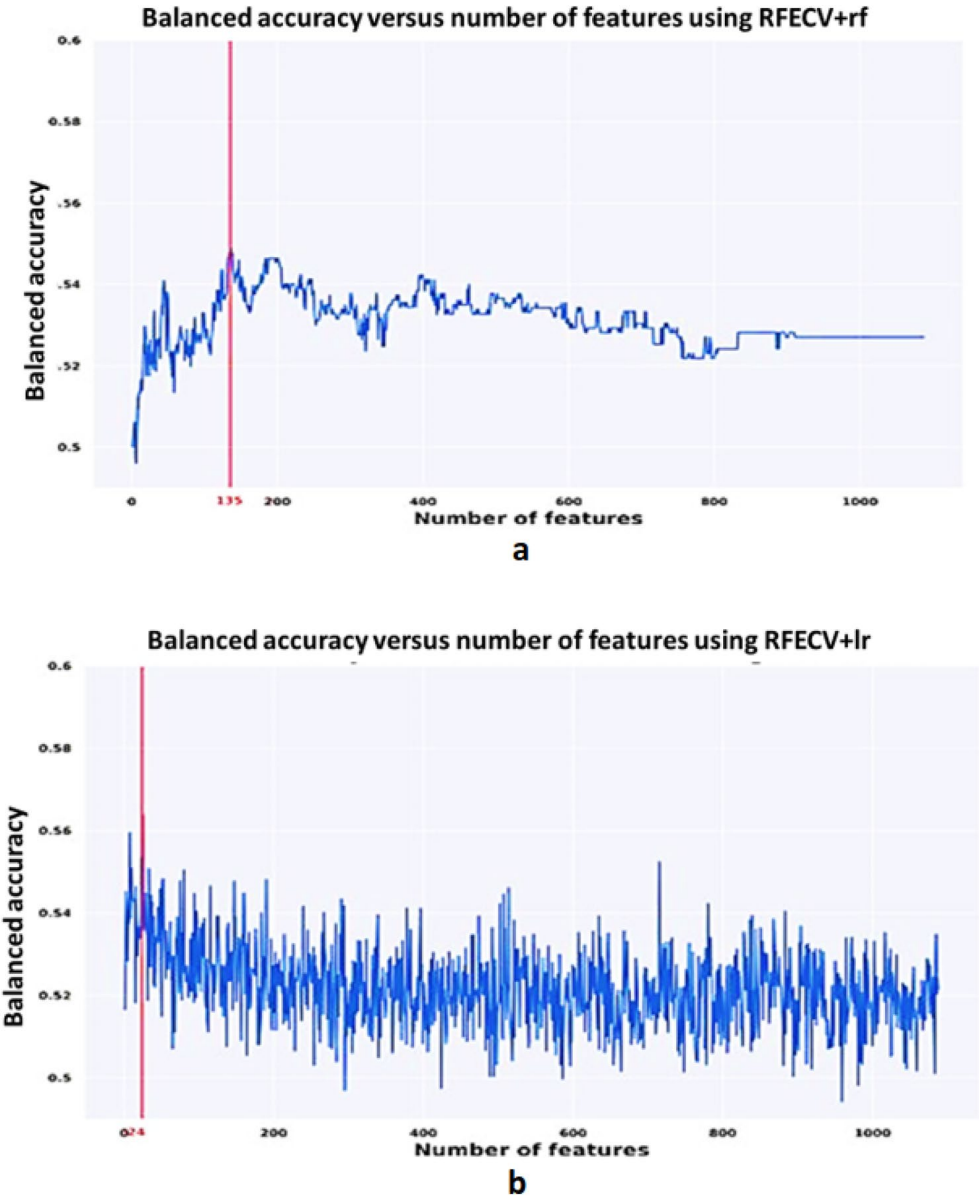
Balanced accuracy versus the number of selected features using each of Recursive Feature Elimination with Cross-Validation (RFECV) classifiers. (**a**) Random forest, and (**b**) Logistic regression.

In this section, we will go through the results which lead us to build multiple neuro atlases for each behavioral disorder. First, features are selected via the Recursive Feature Elimination with Cross-Validation (RFECV) algorithm. Figures 2 and 3 demonstrate the results of each of the RFECV algorithms to select the subset of features which the maximum classification accuracy between sever-cognition ASD and TD. A vertical line indicates the size of the feature set corresponding to the maximum classification balanced accuracy.

**Figure 4:**
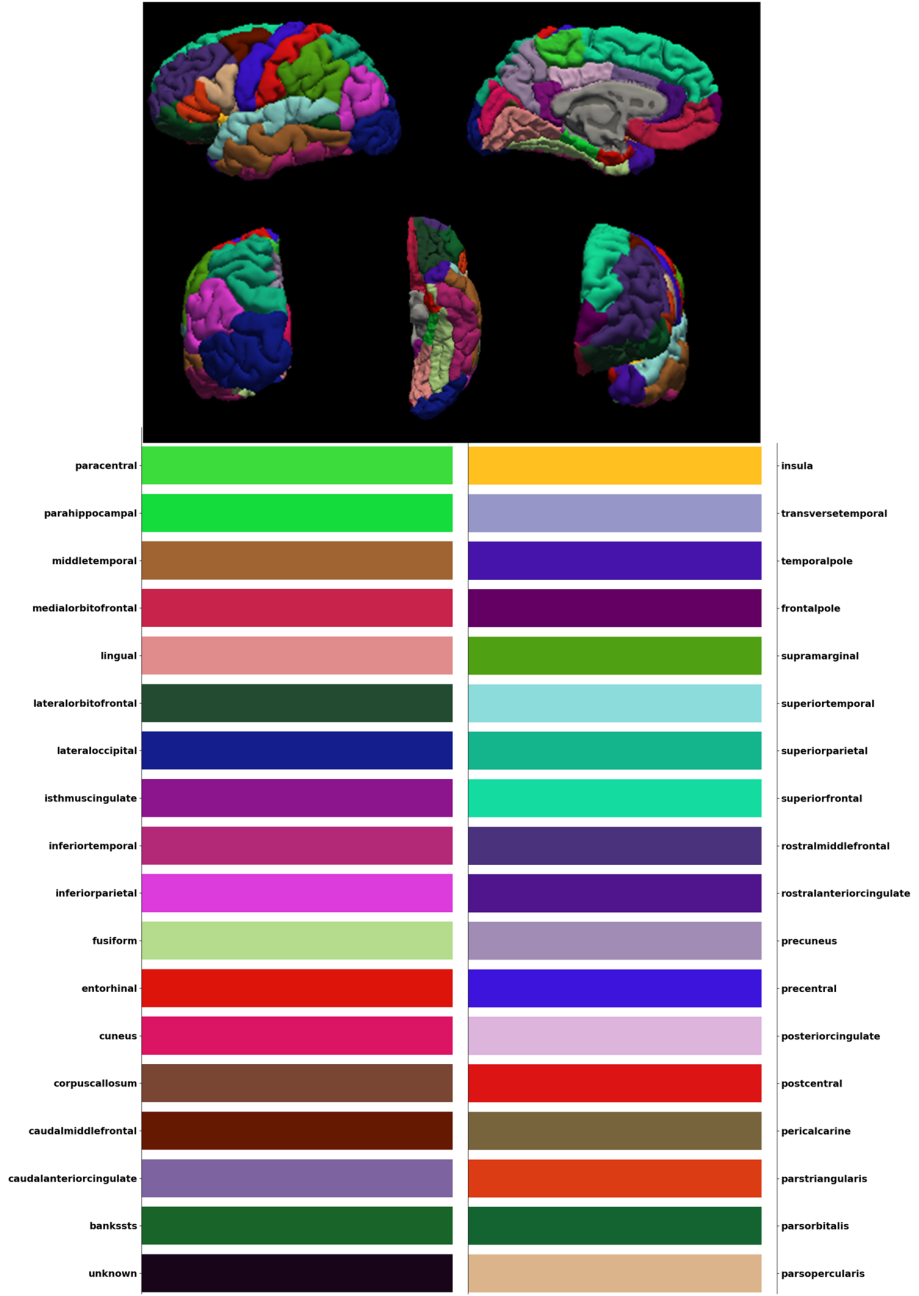
The color-coded brain areas.

**Figure 5:**
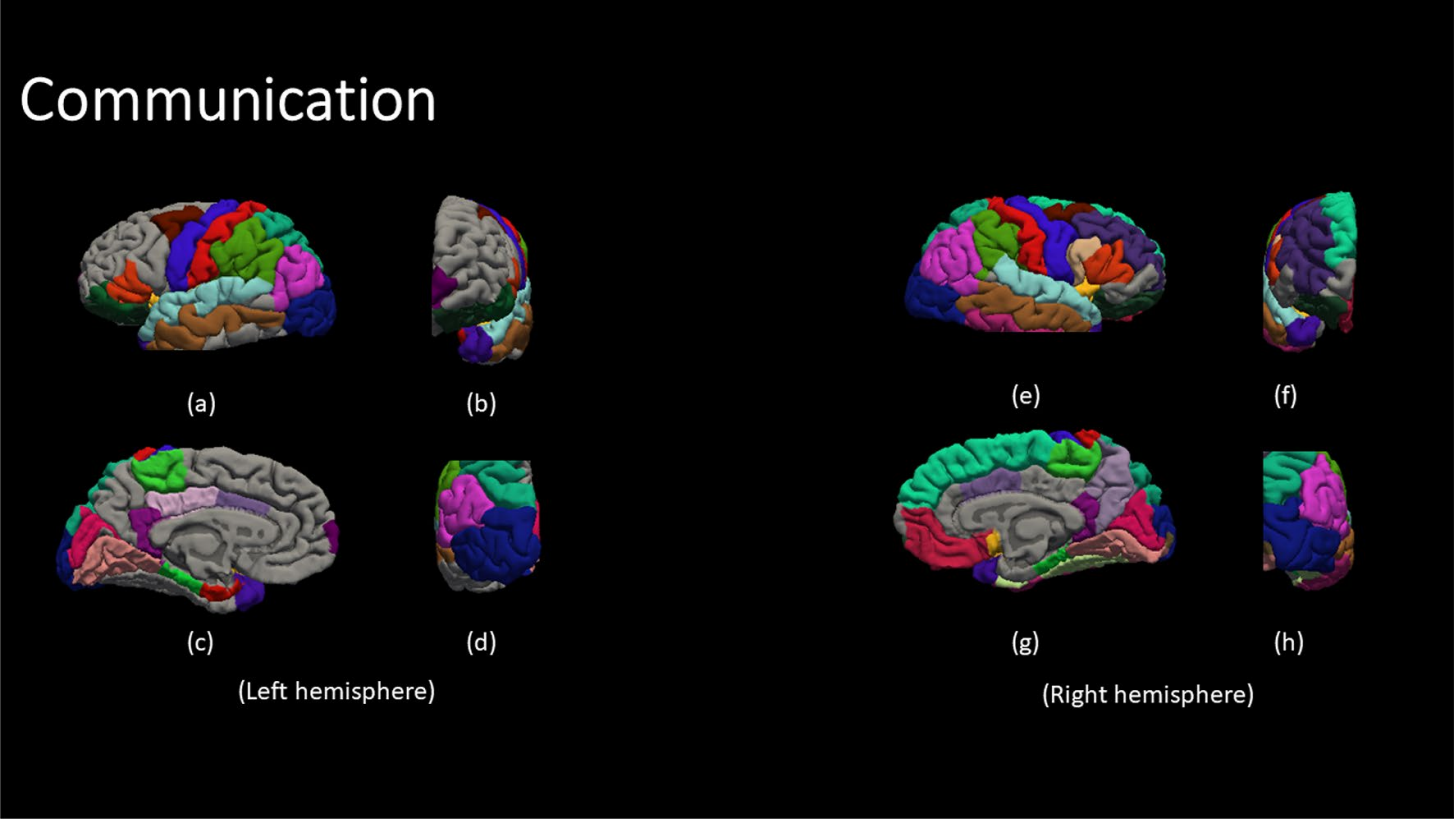
The results for the most frequent brain region overall Severe group for the communication behavioral Report. (**a**,**e**) lateral view, (**b**,**f**) anterior view, (**c**,**g**) medial view, and (**d**,**h**) posterior view.

**Figure 6:**
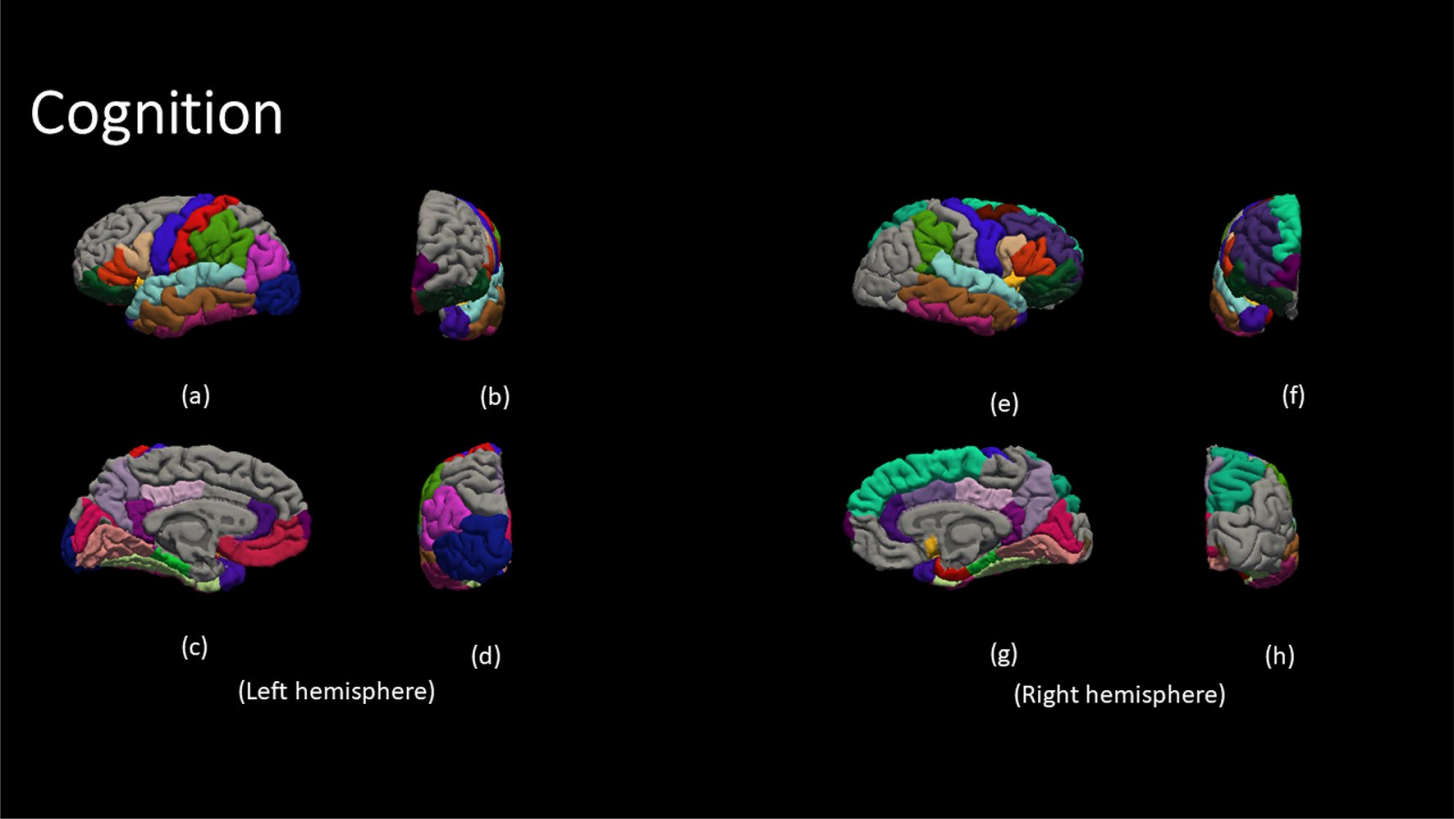
The results for the most frequent brain region overall Severe group for the cognition behavioral Report. (**a**,**e**) lateral view, (**b**,**f**) anterior view, (**c**,**g**) medial view, and (**d**,**h**) posterior view.

**Figure 7:**
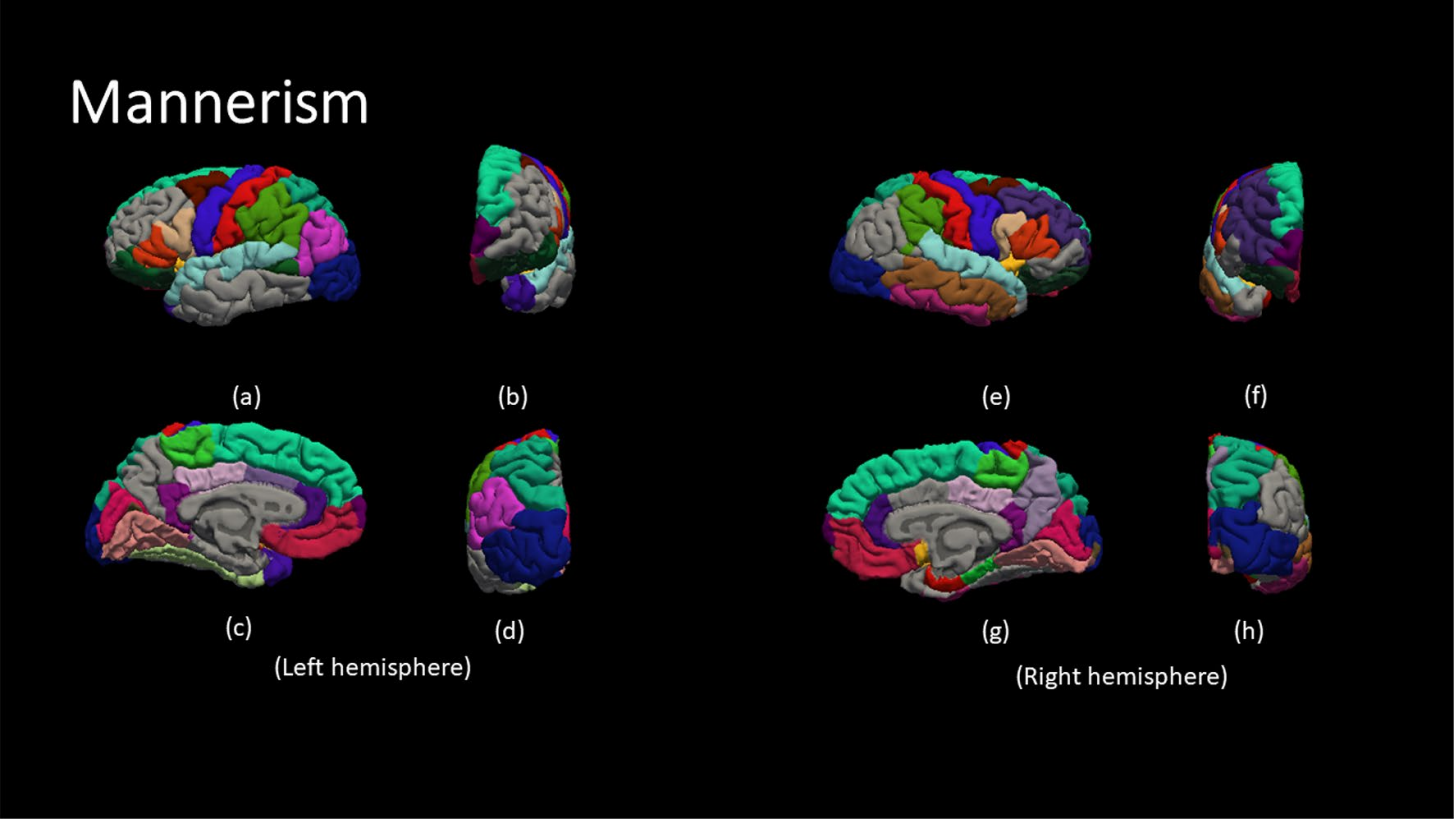
The results for the most frequent brain region overall Severe group for the mannerism behavioral Report. (**a**,**e**) lateral view, (**b**,**f**) anterior view, (**c**,**g**) medial view, and (**d**,**h**) posterior view.

**Figure 8:**
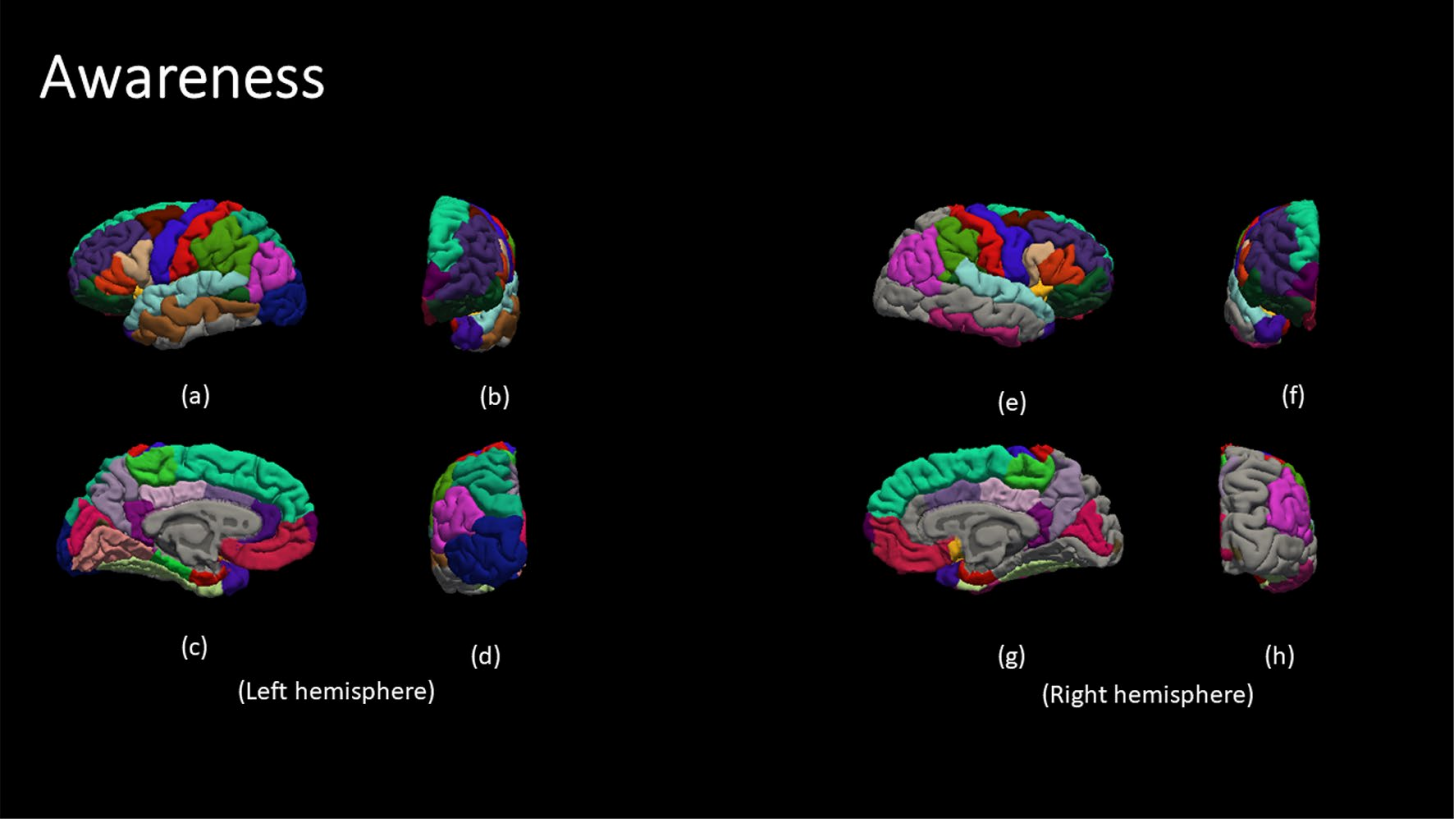
The results for the most frequent brain region overall Severe group for the awareness behavioral Report. (**a**,**e**) lateral view, (**b**,**f**) anterior view, (**c**,**g**) medial view, and (**d**,**h**) posterior view.

**Figure 9:**
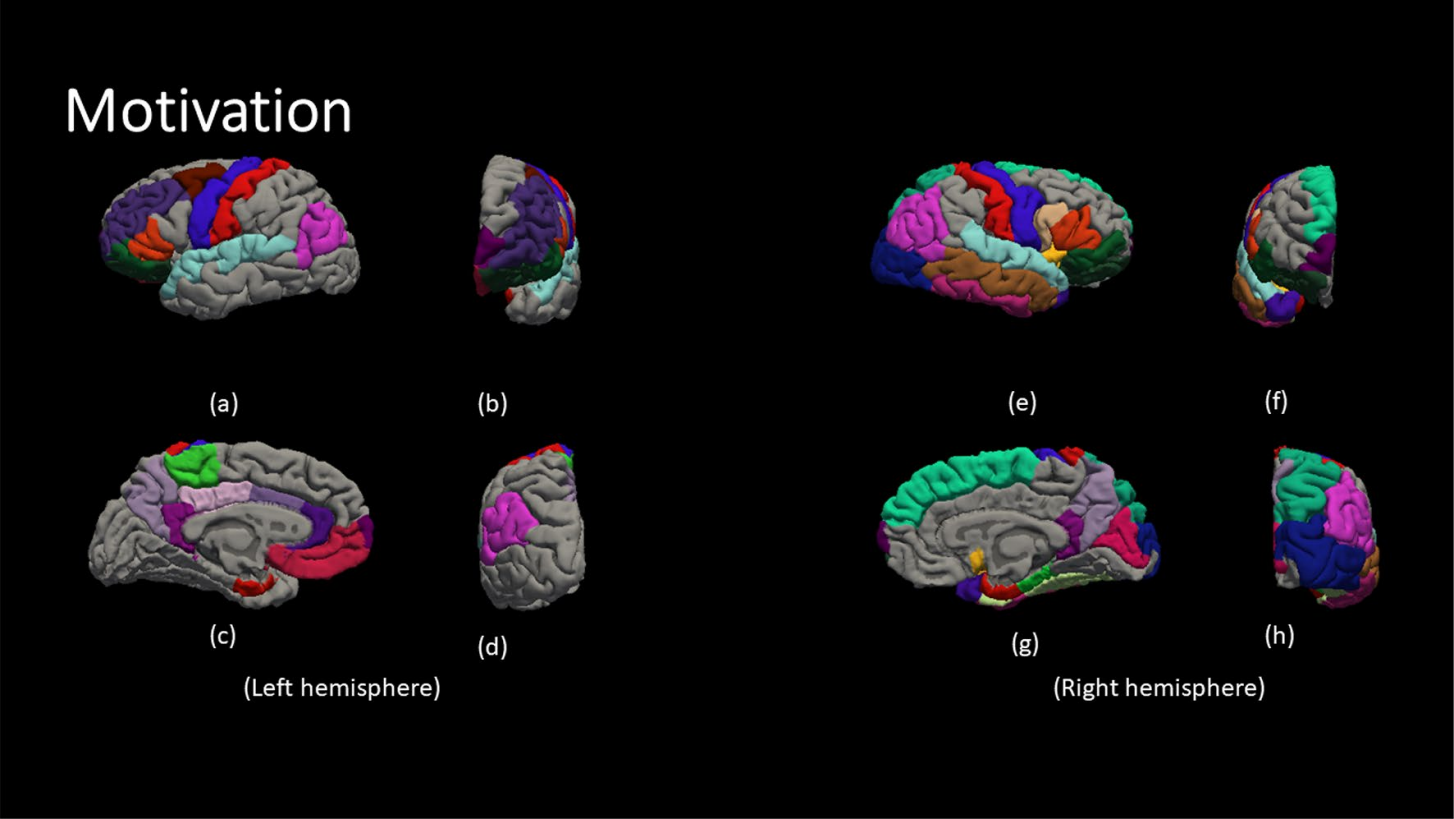
The results for the most frequent brain region overall Severe group for the motivation behavioral Report. (**a**, **e**) lateral view, (**b**,**f**) anterior view, (**c**,**g**) medial view, and (**d**,**h**) posterior view.

Figure 4 demonstrate the color code of each brain region defined by DK atlas. The color codes are the standard color codes defined by Freesurfer. Figures 5, 6, 7, 8 and 9 demonstrate the neuro-atlases that define the sets of brain regions associated with different behavioral domains, i.e., communication, cognition, mannerisms, awareness, and motivation domains.

### ML classifiers

The results introduced in this section are for the experiments that yielded the maximum classification performance between TD and each of the severity-behavioral groups. Unlike the previous section, the models are solely based on their performance on the hold-out set without performing any statistical analysis. The selected features, which are used as the input, for each of the selected ML models are not introduced in this section. Since it is difficult to assume the generalizability of any single experiment, we did not weigh the input features to those models as much as the neuro-atlases which are selected based on statistical significance across all 55 experiments.

Each of those models outputs a probability that a given subject belongs to a severity-behavioral group. Table 1 shows the classification accuracy of each of the selected ML models.

**Table 1:** The behavioral classification results of the top-performing classifiers.

Severity	Behavior	RFECV	Classifier	# features	Bacc	F1
Mild	Awareness	lsvm	svm	183	0.98	0.92
	Communication	lsvm	svm	70	0.99	0.99
	Cognition	lsvm	lsvm	96	0.99	0.95
	Motivation	lsvm	svm	101	0.97	0.85
	Mannerism	lsvm	lsvm	137	0.99	0.99
	Total	lsvm	lsvm	130	0.99	0.99
Moderate	Awareness	lsvm	lsvm	240	0.92	0.8
	Communication	lsvm	lsvm	132	0.96	0.88
	Cognition	lsvm	lr	191	0.98	0.94
	Motivation	lsvm	lsvm	212	0.96	0.91
	Mannerism	lsvm	svm	184	0.98	0.94
	Total	lsvm	lsvm	167	0.97	0.91
Sever	Awareness	lsvm	lr	154	0.93	0.82
	Communication	lsvm	svm	241	0.91	0.84
	Cognition	lsvm	lsvm	254	0.95	0.90
	Motivation	lsvm	lsvm	166	0.96	0.91
	Mannerism	lsvm	lsvm	172	0.95	0.91
	Total	lsvm	lsvm	213	0.92	0.86

Table 2 shows the summary statistics of each severity group overall behavioral groups. The summary statistics are provided for the number of input features for each ML model, hold-out set balanced accuracy, and hold-out set F1-score.

**Table 2:** Severity classification summary statistics.

Severity	# Features (mean ± std. dev.)	Bacc (mean ± std. dev.)	F1 $(mean \pm std)$
Mild	$119.5\pm 39.4$	$0.99\pm 0.01$	$0.95\pm 0.05$
Moderate	$187.6\pm 31.1$	$0.96\pm 0.02$	$0.90\pm 0.05$
Sever	$200\pm 42$	$0.94\pm 0.02$	$0.87\pm 0.03$

Table 3 shows the summary statistics of each behavioral group overall severity groups. The summary statistics are provided for the number of input features for each ML model, hold-out set balanced accuracy, and hold-out set F1-score.

**Table 3:** Behavior classification summary statistics.

Behavior	# Features (mean ± std. dev.)	Bacc (mean ± std. dev.)	F1 (mean ± std. dev.)
Awareness	$192.3\pm 41.7$	$0.95\pm 0.03$	$0.85\pm 0.06$
Communication	$147.6\pm 86.6$	$0.96\pm 0.04$	$0.91\pm 0.07$
Cognition	$180.33\pm 79.5$	$0.97\pm 0.01$	$0.93\pm 0.03$
Motivation	$159.6\pm 55.7$	$0.97\pm 0.003$	$0.89\pm 0.03$
Mannerism	$164.33\pm 24$	$0.98\pm 0.02$	$0.95\pm 0.04$
Total	$170\pm 41.5$	$0.96\pm 0.04$	$0.93\pm 0.06$

## Discussion

In the proposed study, we focus on two aims: (i) build an ML pipeline that mimics the clinical diagnosing process of ASD by classifying ASD in terms of the severity of behavioral domains of the SRS module, and (ii) define behavioral neurocircuits that affect alteration in the subject’s behavior according to SRS module. In the following subsection, we will discuss the findings of each of the aforementioned three aims, and how this work takes a leap toward an objective diagnosis of psychological disorders.

As we previously explained, behavioral classification is meant to serve two tasks: (1) Find the morphological cortical features that may affect the behavior of a subject, and (2) Train an ML model to classify the severity of a subject’s behavior based on the selected morphological cortical features. RFECV with four kernels (LR, RF, LGBM, and LSVM) are employed to find the subset of cortical morphological features that maximize the balanced accuracy score of classifying the severity of ASD according to every SRS behavioral module. Each of the RFECV four kernels is initialized with the default parameters as specified in scikit-learn package, therefore, we are not expecting to achieve the highest possible classification accuracy while searching for the subset of morphological features, however, we are looking for the subset of morphological features that will correspond to the maximum classification accuracy given the utilized kernel. Thus, the maximum accuracy observed in Figs. 2 and 3 are far below those in Table 3. This is due to the fact that the ML models utilized at the end of the behavioral classification step are optimized using a random search grid in a different feature space with lower dimensionality.

By the end of behavioral classification, we have an optimized ML model trained on a selected subset of features that results in the highest classification accuracy between TD and a severity-behavioral ASD group. Although this subset of morphological features can be thought of as the atlas/neurocircuit, which affects this behavior trait, we still do not have any statistical significance to support our findings. Therefore, we decided to create the behavioral classification optimization step at which we repeated the behavioral classification 51 times while randomly sampling the training-validation dataset every time in order to confirm the findings on different subsets of data. The statistically significant morphological cortical features, which demonstrated significance at $\alpha =0.001$, are considered to be the neuro-atlas that defines the behavioral group at a given severity level for ASD. We aggregated the behavioral group findings over different severity levels to get a cortical atlas that defines the behavioral spectrum of ASD, as shown in Figs. 5, 6, 7, 8 and 9.

The behavioral classification defines and classifies ASD as a behavioral spectrum. In the proposed study, that behavioral spectrum is defined in terms of SRS module, however, it can be defined in terms of any other ASD diagnostic reports such as ADI-R, or ADOS. Behavioral classification is a standalone CAD system that places a subject into the ASD spectrum. ASD spectrum is thought of as a multidimensional space in which each dimension represents a severity of a given behavioral trait, and the diagnosis of a subject depends on the location of that subject within that space.

The numerical results of the behavioral classification are summarized in Tables 1, 2, and 3. Table 1 demonstrates the results of the top-performing severity-behavior models which are used to predict the probability of a subject being within that severity-behavior group vs TD. Table 2 demonstrates the mean, and the standard deviation of the number of features, the balanced accuracy score, and the F1-score of classifying each severity against TD overall behaviors. We assumed that the easiest to classify would be severe vs TD, since it is the one, we assumed, with the highest contrast. However, the behavioral classification step requires more number features to successfully classify severe ASD vs TD with a mean bacc of 94%. Moreover, mild ASD vs TD is the easiest to classify utilizing only 120 features on average, across all behavior groups, with bacc of 99%. The rationale behind this counter-intuitive result is the fact that a severe ASD subject would have more cortical morphological alteration, which will lead the feature selection step to select more features that discriminate between TD and severe ASD. With more features, an ML model requires more number of subjects to be able to achieve higher cross-validation results. From a different perspective, we hypothesize that severe ASD is merely a label for heterogeneous ASD traits on the spectrum. Therefore, a clinical solution would be to further study severe ASD and split it into further simpler traits that are more homogeneous and can be described with fewer cortical features. Table 3 demonstrates the mean, and the standard deviation of the number of features, the balanced accuracy score, and the F1-score of classifying each ASD behavioral group against TD overall severity. Table 3 demonstrates that mannerism has the highest classification accuracy on average followed by cognition, and motivation, followed by communication, and total and eventually awareness. Again we observe similar results to those in Table 2, the greater number of features selected to classify behavioral ASD group and TD, the less the mean classification accuracy.

Table 4, covers the significance of the detected brain regions, in the behavioral classification step, according to the literature to be associated with specific to ASD and SRS. For each behavioral trait, we demonstrate how the associated brain regions are also nominated in the literature as relevant to that behavior. The striking part of the behavioral mapping atlas is that many regions contribute to the category subscores on the SRS-2 including awareness, cognition, mannerisms, communication, and motivation (Table 4). Very few regions just contribute to 2 or fewer subscores in our results. Thus, the high sensitivity and accuracy of this classification algorithm stems from its ability to integrate abnormal MRI parameters and mapped behavioral characteristics over the entire brain (Table 1, 2 and 3). At the individual (though not the subject of this paper), it is likely that a few MRI parameters from particular brain regions contribute to the elevated severity subscores on SRS-2 and thus classifying an individual as not only belonging to ASD as a disease but also clinical severity class of mild, moderate, or severe ASD as defined on the SRS-2.

The brain regions of our results (Table 2) suggest that the classifier affects primary (auditory, visual) sensory-motor regions, secondary brain networks such as working memory, and tertiary/meta networks (interactions of secondary networks) that mediate complex tasks such as goal-directed behavior. Here we discuss those networks and, their relation to the SRS. Our paper is novel in that no literature paper describes the comprehensive relationship between clinical severity, all brain regions, and ASD. Weerasekera et al.^33^ looked at 58 subjects of the ABIDE dataset and found that left amygdala total volume but not full-scale IQ in ASD subjects was negatively correlated with the SRS social cognition subscore. Since emotional learning and memory modulation are primary amygdala functions, SRS-2 subscore in our dataset (Table 4) were accurately found to classify ASD vs TD including anatomical and functionally connected areas [caudal ACC, left and right anterior plus rostral ACC, right and left insula, left and right temporal pole, left and right superior frontal region (DLPFC), left and right middle frontal cortex, left and right MTG, and left and right frontal pole]. A study of 101 children with ASD (Cheng et al.^34^) discerned the relationship between obese/overweight, gray matter volume (GMV), and SRS-2 Total score. The GMV of the left superior frontal cortex mediated part of the positive correlation (36.6% of the variance) between obese/overweight parameters and the SRS-2 Total score. Similarly, the left and right superior frontal cortex (DLPFC) mapped to the social cognition, social motivation, and autistic mannerisms subscores of the SRS-2 (Table 4). Another study of two separate age and sex-matched cohorts (Plitt, Barnes, Martin^35^ study) including one from the ABIDE dataset (89 ASD + 89 TD) suggested that SRS and resting functional state MRI (rs-MRI) could be good classifiers in ASD. This study did not incorporate the two together as in this report. Rs-MRI predicted regions involved social function and task control were good classifiers similar to this report (Table 4) denoted with an asterisk (Insula*, Ventromedial prefrontal cortex, anterior/middle/posterior cingulate cortex*, posterior fusiform gyrus, posterior superior temporal sulcus*, temporal parietal junction, intraparietal sulcus*, and inferior and middle frontal gyrus*). Finally Chen *et al*. (2019) looked at a cohort of age/sex-matched 21 ASD subjects and 23 TD subjects who had a modest negative correlation ($-0.33$ to $-0.42$) between the default mode network (DMN) and social awareness, social cognition, social communication, social motivation, and autistic mannerisms as a classifier. Similarly, these DMN regions (posterior cingulate gyrus*, left occipital cortex*, right MTG) except the medial PFC mapped onto the SRS-2 subscores noted in Table 4 and add to a stronger more accurate classier (Tables 1, 2). In overview, this study confirms but greatly extends the use of SRS-2 and MRI in classification algorithms with the addition of creating a novel machine learning paradigm.

**Table 4:** The significant brain regions, in the behavioral classification step, according to the literature to be associated with specific to ASD and SRS. Letters in the third column denote the following citations: (A) Weeraskera et al.^33^, (B) Cheng et al.^34^, (C) Plitt, Barnes, and Martin^35^, and (D) Chen et al.^36^.

Brain region	SRS subscore	SRS, MRI, and Classification in Literature	Normal function
Caudal Anterior Cingulate Cortex (ACC)	Cognition, Mannerisms	A	Emotional regulation, attention, cognitive control, pain perception, information integration^37^
Left Caudal Middle Frontal Gyrus (Broca)	Awareness, Communication, Mannerisms	C	Understanding & Execution of language (speech, written), nonverbal communication^38^
Right Caudal Middle Frontal Gyrus	Awareness, Cognition	C	Math & visual-spatial operations, selective attention, problem solving, and decision making^38^
Left Insula	Motivation, Awareness, Mannerisms, Cognition	C	Negative emotions, attention, decision making, memory, language social cues^39^
Right Insula	Awareness, Motivation, Mannerisms, Cognition	C	Positive emotions, self awareness, reward processing, decision making^39^
Left and Right Anterior ACC	Awareness, Communication, Cognition, Motivation	A	Attention, conflict resolution, empathy, goal-directed behaviors, memory, emotion-pleasure-desire regulation, repetitive behaviors, social/emotional processing^40^
Left Lateral Occipital Region	Awareness, Mannerisms, Cognition	D	Quick recognition of objects, their shapes and forms^40^
Left and Right Lingual Gyri of Occipital Lobes	Awareness, Mannerisms, Cognition		Visual memory, recognition of faces and objects^41^
Left and Right Middle Temporal Gyrus (MTG)	Cognition, Awareness, Communication	A, D	Attention and navigation of environment, memory, attention, spatial processing, language processing + prosody^42^
Left and Right Posterior Cingulate Cortex (PCC)	Communication, Mannerisms, Cognition, Awareness, Motivation	C, D	Attention, working memory, executive control, self-awareness, motivation, goal-directed behavior^43^
Left and Right rostral Anterior Cingulate Cortex (rACC)	Awareness, Motivation, Mannerisms, Cognition	A, C	Emotional regulation, attention, decision making, emotional regulation, goal-directed behavior^44^
Left and Right Temporal Pole (LTP and RTP)	Communication, Cognition, Awareness, Motivation	A	Object meaning, language, nonverbal cues, social behavior, attention, memory, emotional regulation, reward processing^45^
Left and Right Transverse Temporal Region (LTT, RTT)	Communication, Motivation		Auditory processing & interpretation, perception of auditory stimuli (speech, music), auditory integration with other senses^46^
Left and Right Superior Frontal Region (DLPFC)	Cognition, Motivation, Mannerisms	A, D	Working memory, emotional regulation, control of behavior^47^
Left and Right Superior Parietal region	Awareness, Mannerisms	C	Working memory representation, attention, fine motor movements, object perception, and manipulation^48^
Left and Right Superior Temporal Region (LST and RST)	Communication, Awareness, Motivation, Mannerisms	C	Auditory/speech processing, auditory regulation of movement & motivation, sensory input integration, music perception, attentional focus/awareness of environment^49^
Left and Right Frontal Pole (LFP and RFP)	Awareness, Communication, Mannerisms, Motivation	C	Consciousness, memory, attention, language-related behavior, sensory processing, regulation of voluntary motor & goal-directed behavior^50^
Left and Right Rostral Middle Frontal Cortex (LMF & RMF)	Awareness, Communication, Mannerisms	A	Emotion regulation, working memory, cognitive strategies of emotion regulation, temporary storage and manipulation of information, communication, allocating attention resources to language^51^

In summary, our AI-based model was able to accurately differentiate between the functionalities of specific brain regions, such as the left and right caudal middle frontal regions, in the classification of behavioral severity of Autism Spectrum Disorder is a significant advancement in the field. The model has found that the left caudal middle frontal region is linked to mannerism, awareness, and communication behavioral domains, while the right caudal middle frontal region is primarily associated with awareness and cognition, making it more associated with analytical processes rather than social processes. This is particularly noteworthy because previous research has also documented a remarkable difference between the left and right caudal middle frontal regions. At the same time, most studies show little to no difference between other left and right brain regions. This shows that our AI model has the ability to accurately identify specific contralateral regional differences or similarities, adding to the validity of the model’s findings.

It is important to note that all behavioral domains are complex and multifaceted processes, and many different brain regions are thought to be involved in different aspects of it. It is also important to note that the field of neuroscience is always evolving and the understanding of the function of different brain regions is still under research. One of the limitations of the proposed study is the absence of a data harmonization protocol devoted to the elimination of the site effect in neuroimaging studies. As was shown by Saponaro et al.^52^, multi-center data collections may suffer the batch effect, which, especially in the case of Magnetic Resonance Imaging (MRI) studies. They should be curated to avoid confounding effects for ML classifiers and masking biases.

## Methods

A novel ML framework (Fig. 1) is proposed in this study. The proposed framework selects morphological features and finds the anomalous neurocircuits associated with every behavior defined in SRS. An ML module, that utilizes the selected morphological features, is utilized to classify subjects to different severity levels within each behavioral group. In order to build the neuroatlases associated with each behavioral category, we repeat the behavioral classification step 51 times, while shuffling the training-validation dataset, to find cortical regions with a discrimination power of statistical significance of $p<0.01$. The proposed framework begins with obtaining sMRI volumes of both ASD and TD subjects from the ABIDE II dataset^32^. The sMRI volumes then undergo preprocessing using Freesurfer v6.0 software^53–56^. The preprocessing consists of three stages, namely intensity normalization, skull stripping, and brain segmentation. Each stage contains multiple substages, which will be explained briefly in the following sections. After preprocessing, the behavioral classification step begins with subjects being categorized according to behavioral modules of SRS, then within each module subjects are labeled according to their standard SRS score either be TD, mild, moderate, or severe. The proposed framework extracts features from each subject’s MRI volumes and summarizes them in eight numerical representations for each morphological feature and brain region. Next, a data matrix and target vector are created for each behavioral module and passed to a feature selection algorithm to select imaging markers. Then, the proposed framework creates dimensional reduced data matrices using the selected imaging markers and target vector and passes them to ML algorithms to identify the best model for classifying ASD and TD subjects.

Figure 1 demonstrates the general block diagram of the proposed framework comprising two parts: the behavioral classification step post-data preprocessing and feature extraction. The first part comprises data preprocessing, feature extraction, and categorizing subjects based on behavioral modules of SRS, we name subjects within each behavioral category as a behavioral group. The second part is the behavioral classification step (see Fig. 1), where each behavioral group is split according to their normalized severity scores into TD ($\le 59$), mild (60–65), moderate (66–75), and severe ($\ge 76$).

### Data

**Figure 10:**
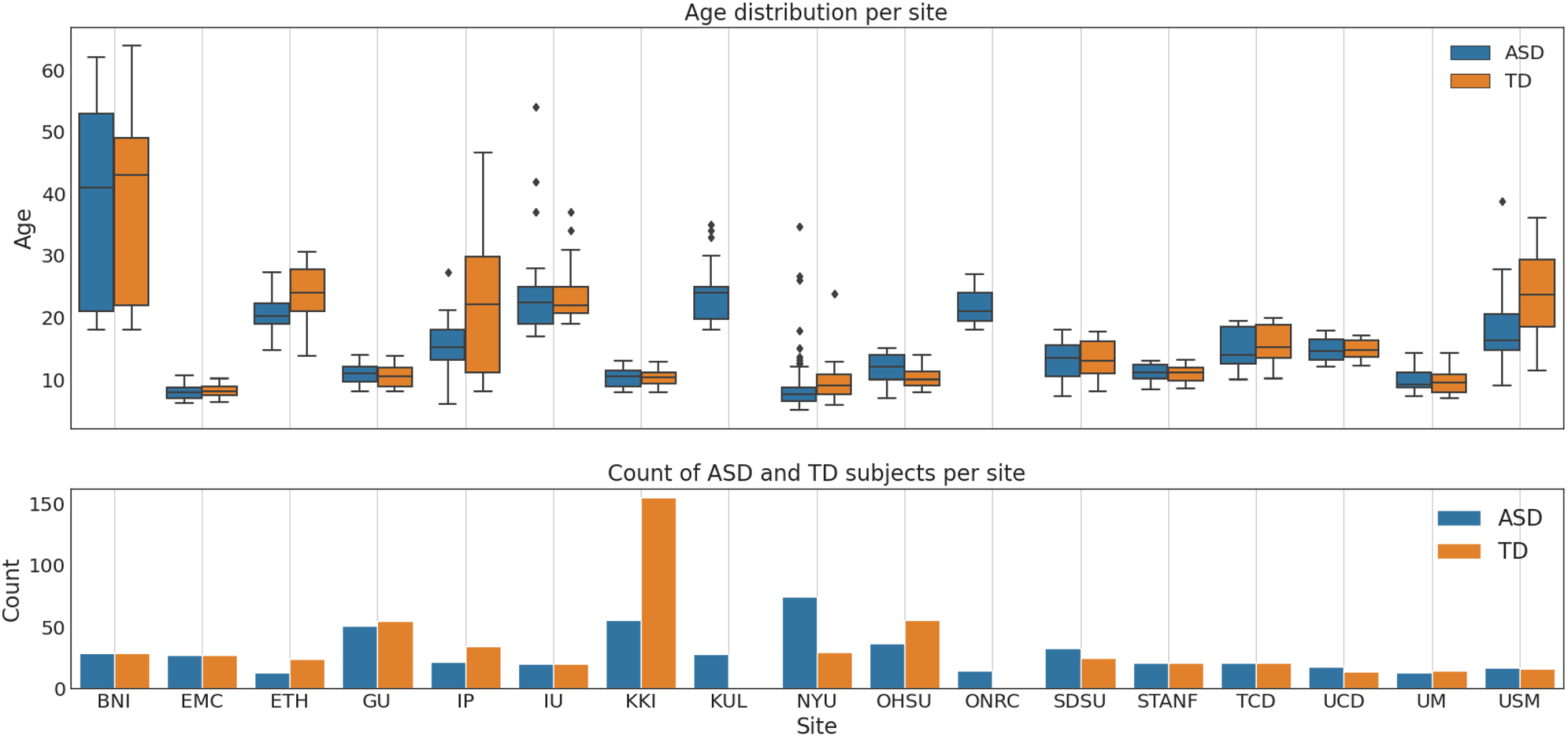
(Top) boxplot of subjects’ age distribution on the vertical axis versus sites’ names on the horizontal axis for each phenotype. (Bottom) the number of ASD and TD subjects on the vertical axis versus the sites’ names on the horizontal.

The online available ABIDE II dataset is utilized in this study. Ethical approval was not required as confirmed by the license attached with the open-access data, since they were previously approved by each site’s local IRB. ABIDE II is the second iteration of the Autism Brain Imaging Data Exchange (ABIDE), which aims to enhance the scope of brain connectomics research in ASD. It is worth noting that the first iteration of ABIDE was ABIDE I, which was released in 2012 with data from 1112 subjects acquired from different clinical sites^32^. ABIDE II dataset comprises 521 individuals with ASD and 593 TD subjects. After preprocessing and quality control, we ended up including 496 individuals with ASD and 542 TD subjects from a total of 17 sites. Figure 10 describes the summary statistics of the cohort included in this study. To ensure the successful preprocessing of all subjects, a quality control process was implemented. All subjects whose scans failed segmentation or exhibited missing cortical features in the output of Freesurfer were excluded.

**Figure 11:**
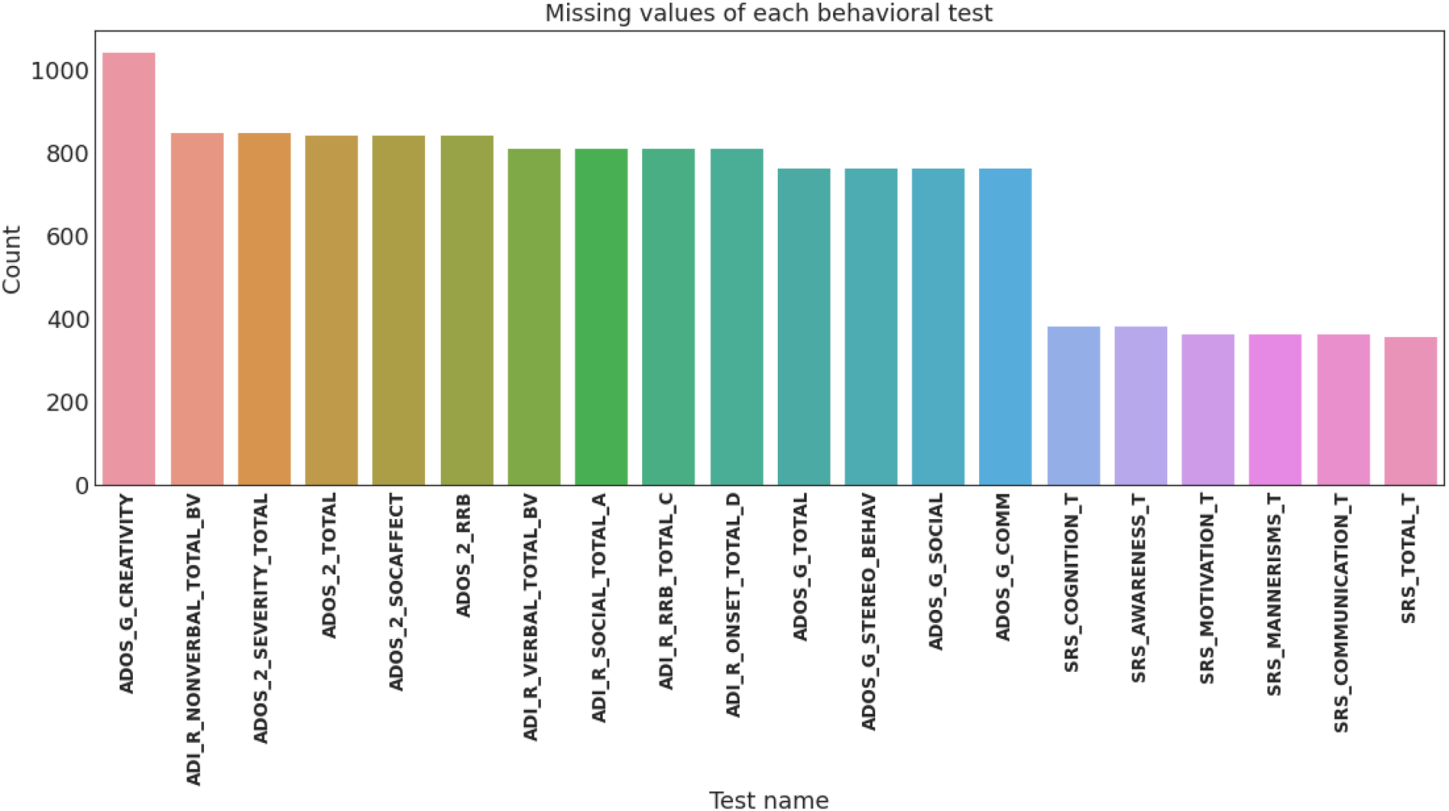
Bar plot demonstrating the number of missing values for each behavioral module included in the ABIDE II dataset.

All included subjects are then grouped into behavioral groups according to the SRS. Figure 11 demonstrates the missing values of each behavioral module provided by ABIDE II dataset such that each bar represents the number of missing values for that given behavioral test. The shortest six bars belong to the SRS tests. Figure 11 is the main motive behind selecting SRS to be the behavioral ground truth for this study since it possesses the least number of missing values.

**Figure 12:**
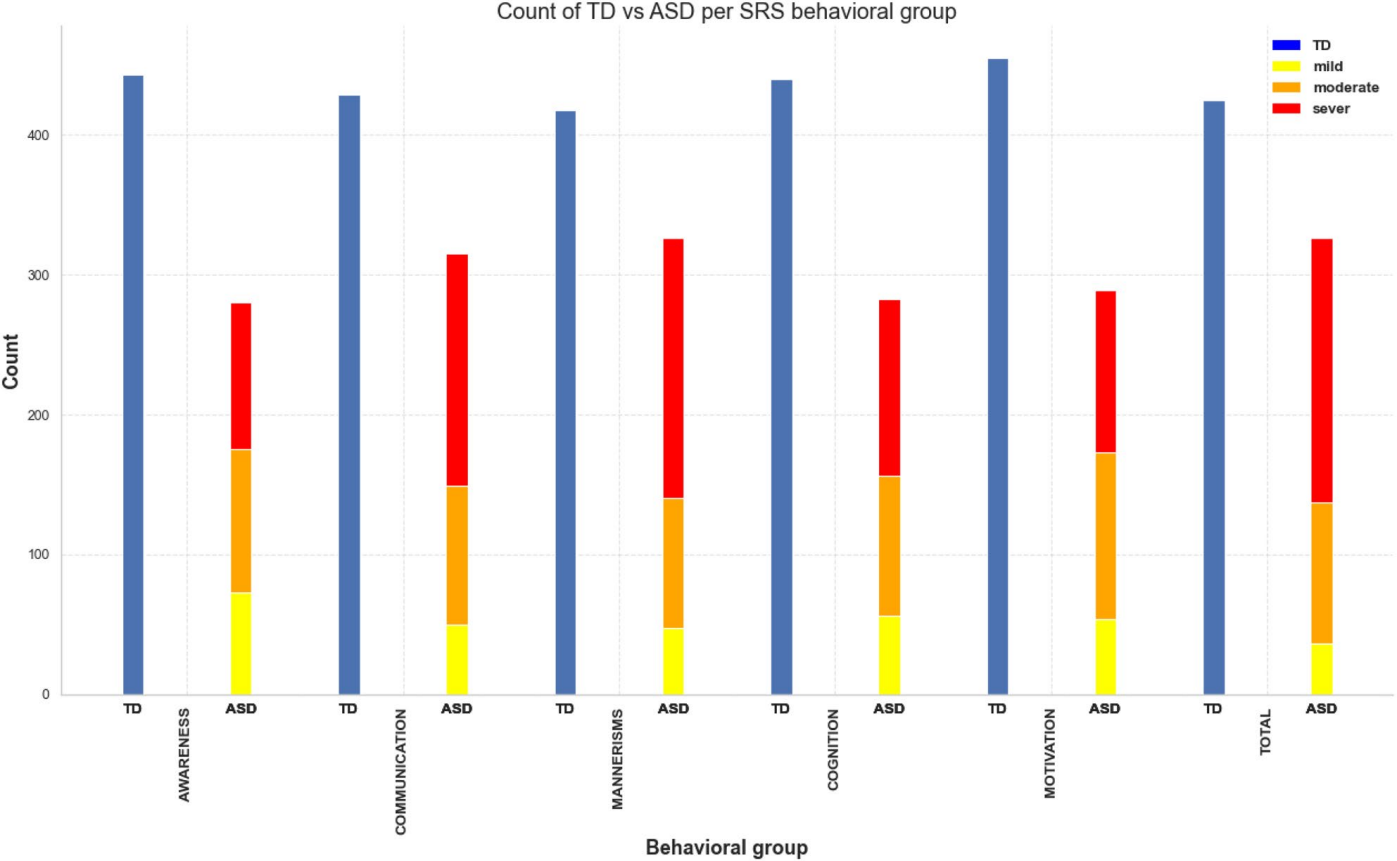
Bar plot demonstrating the counts of TD vs ASD severity group stacked on each other for each behavioral category.

After grouping the subjects into behavioral groups, each group is considered as an independent dataset to be utilized in solving a multi-class classification problem between TD and three levels of severity of ASD. Figure 12 demonstrates the counts of TD and different severity levels of ASD within each behavioral category. As it is obvious, the data are imbalanced, especially with respect to discrimination of mild vs TD. In later sections, we will discuss how we proposed to achieve an unbiased model.

In the following section, we will briefly cover the preprocessing pipeline applied to the sMRI volumes of each subject to extract the cortical morphological features.

### Pre-processing

**Figure 13:**
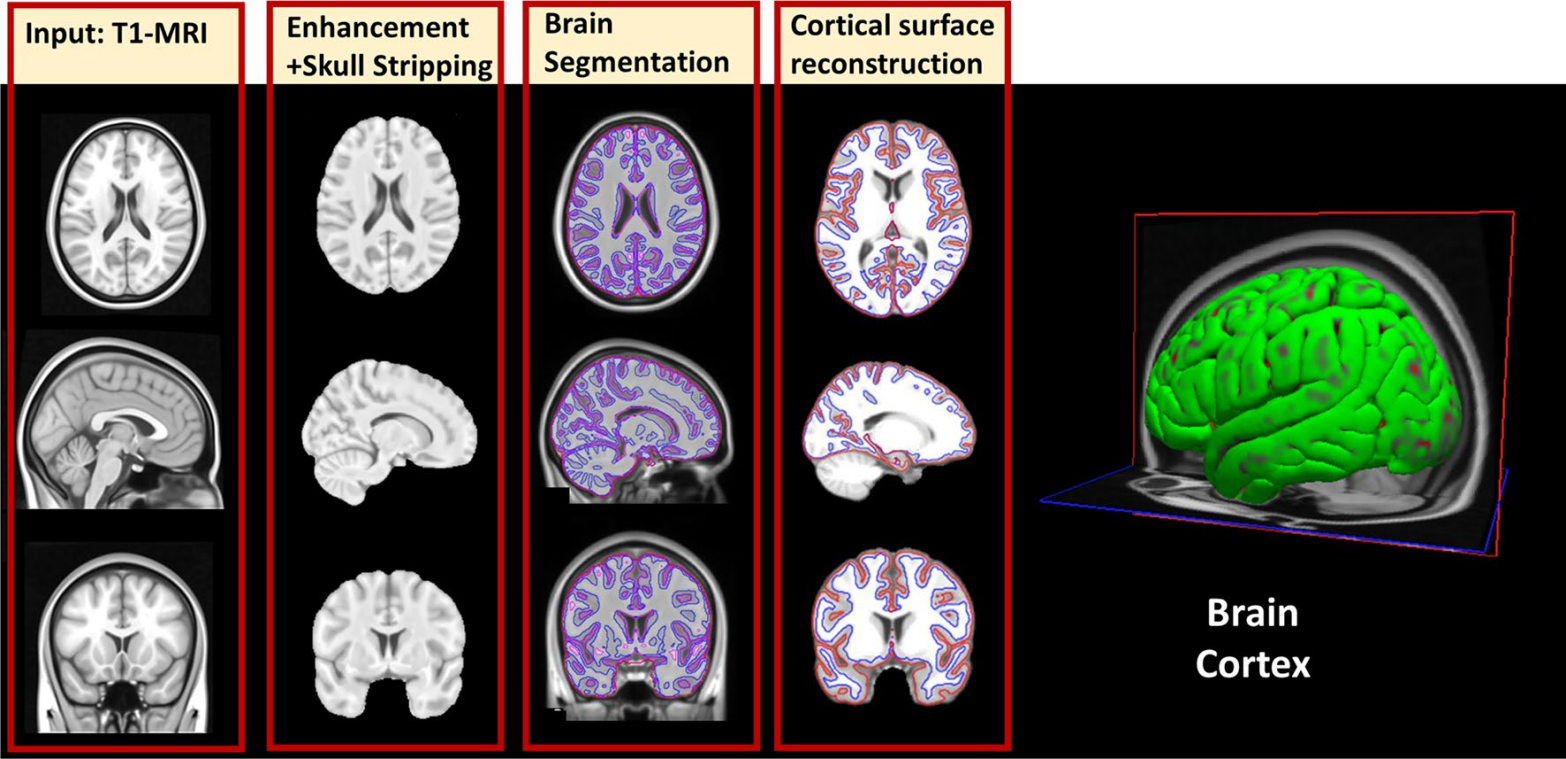
The pipeline of the preprocessing step using Freesurfer v6.0. There are three stages involved in preprocessing, namely: (**i**) normalizing intensity and stripping the skull, (**ii**) segmenting the brain, and (**iii**) Cortical surface reconstruction.

Preprocessing is an essential step to minimize inter-subject variability that can arise from data acquisition, various scanners used, artifacts, or the presence of non-brain tissues in MRI scans, as illustrated in Fig. 13. A sequence of steps is performed to extract the morphological features from the sMRI scans. Those steps comprise (1) normalizing the intensity of each image of the sMRI scans, (2) extracting the brain out of the images, (3) stripping the skull to avoid segmentation errors, (4) Segmenting different brain regions, (5) labeling areas, (6) gray-white matter boundary tessellation, (7) inflating surface, (8) registering the inflated surface to a spherical atlas, and eventually, parcellating the surface of the cortex according to the Deskian-Killiany (DK) atlas.

### Behavioral classification

In this section, we will discuss the proposed framework. As shown in Fig. 1, the input to the behavioral classification step is a matrix containing the values of the morphological features extracted using FreeSurfer for each subject along with a target matrix containing the severity score of each SRS test. The output of the behavioral classification step, as shown in Fig. 1, is an optimized trained classifier and a set of selected features for each behavioral group. In the following sections, we will discuss each step of the behavioral classification in detail.

### Feature extraction

FreeSurfer produces two main outputs. The first output is a set of volumes corresponding to the processing steps from normalization up to segmentation as illustrated in Fig. 1 (bottom right). The second output consists of surfaces that are parcellated according to the DK atlas, which contains morphological feature values at each vertex on a predefined mesh grid covering the cortex.

**Figure 14:**
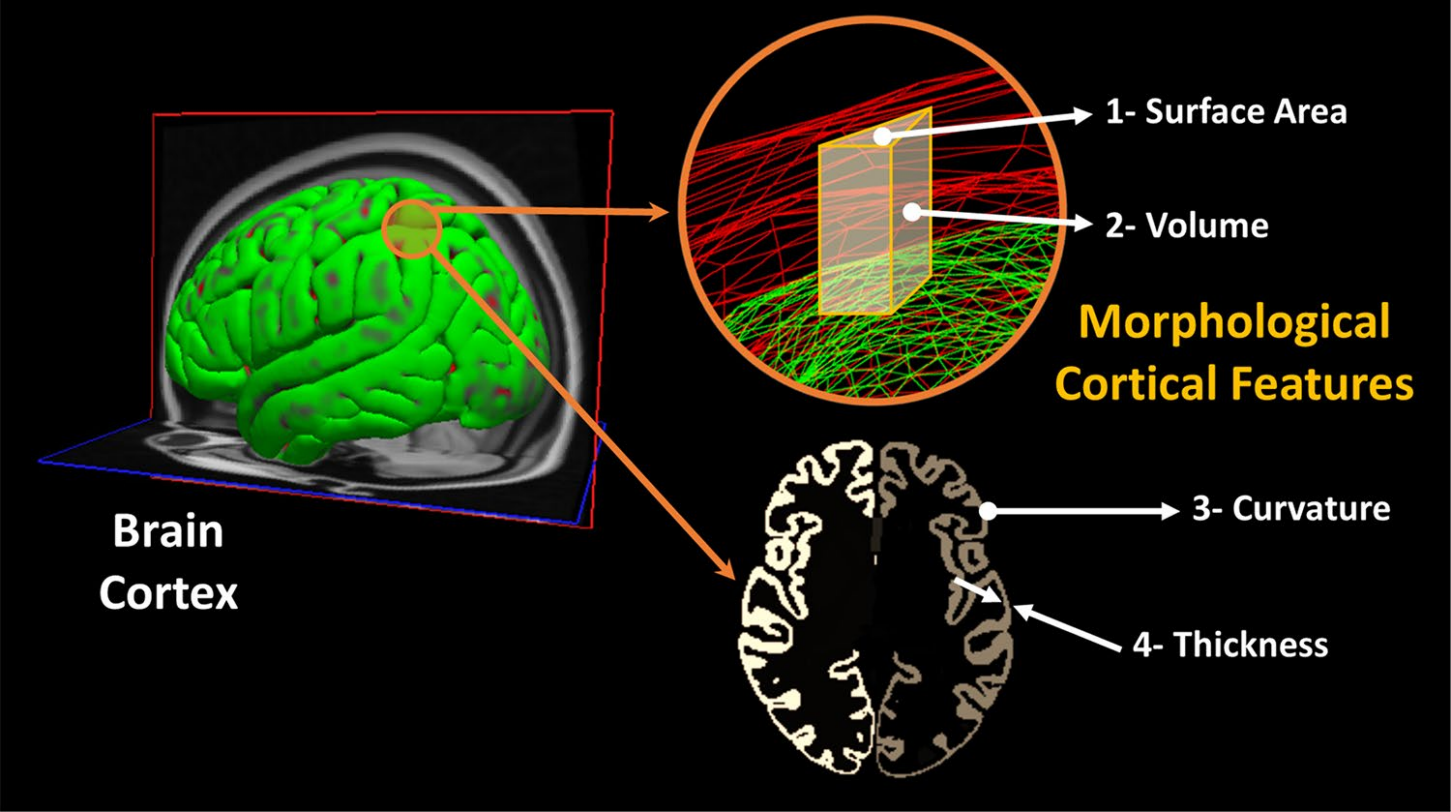
The morphological features extracted by the FreeSurfer from the cortical surface.

The brain of each subject was represented using the following morphological features in this study: (1) surface area, (2) volume, (3) thickness, and (4) curvature, as illustrated in Fig. 14. It is important to mention that thickness is computed as the shortest distance between the gray/white matter boundary and the gray/CSF boundary at each vertex on the tessellated surface^57^. Meanwhile, the curvature is determined as the principal radii reciprocal mean^58^. We calculated the 20th, 40th, 60th, and 80th percentiles for each feature within each brain region of the DK atlas. The main motives behind choosing those four percentiles to represent each morphological feature of each brain region instead of mean and standard deviation, which are already provided by FreeSurfer, (1) to exclude outlier and preprocessing errors within each brain region, and (2) to have more samples representing the distribution of the morphological values within each brain regions. The brain parcellation using the DK atlas divides the brain into 68 regions, with 34 regions on each hemisphere, resulting in a feature vector of $68 \times 4 \times 4 = 1088$ elements representing each subject.

For each of the behavioral groups, we subdivide the ASD subjects according to their severity into mild, moderate, and severe. The main point behind this dividing ASD within each behavioral group is to address the magnitude of the disorder.

For each of the behavioral groups, a data matrix $D_L$ is created such that $D_L = \{d_t: d_t \in R^{M\times 1088}\}$ each corresponding to one of the sites; $d_t$ denotes the data matrix corresponding to behavioral group *t* where $t \in \{$communication, mannerism, motivation, cognition, awareness, total$\}$. Total is an SRS behavioral module denoting the overall score of a subject. Each $d_t$ has the size of $M\times 1088$, such that *M* denotes the number of subjects within site t; sequentially, $y_t$ denotes the diagnosis vector corresponding to site *t*, and $y_L$ denotes the set containing all the $y_t$ for all sites. Equation (1)shows the shape and the symbolic representation of the data matrix and the target vector of each behavioral group.

1 $$\begin{aligned} d_t=\begin{bmatrix}f_{1,1}&{}f_{1,2}&{}\cdots &{}f_{1,1088} \\ f_{2,1}&{}f_{2,2}&{}\cdots &{}f_{2,1088} \\ \vdots &{} \vdots &{} \ddots &{} \vdots \\ f_{M, 1}&{}f_{M,2}&{}\cdots &{}f_{M, 1088}\end{bmatrix},\;\;\;\;\;\;\;\;\;\;\;\; y_t=\begin{bmatrix}y_1\\ y_2\\ y_3\\ \vdots \\ y_{M}\end{bmatrix},\;\;\;\;\;\;\;\;\;\;\;\; D_L = \begin{bmatrix}d_1\\ d_2\\ d_3\\ \vdots \\ d_{6}\end{bmatrix},\;\;\;\;\;\;\;\;\;\;\;\;y_L = \begin{bmatrix}y_1\\ y_2\\ y_3\\ \vdots \\ y_{6}\end{bmatrix} \end{aligned}$$

### Feature adjustment and normalization

Given the literature suggesting an influence of age on ASD brain morphology^59^, the proposed work addresses this by adjusting morphological features for both age and sex. To achieve this, regional metrics of volume (*V*) and surface area ($S_a$) were computed, utilizing cortical growth curves from Coupé et al.^60^. Specifically, let $V_s(a)$ represent the average volume of cortical grey matter in individuals of sex *s* and age *a*. Subsequently, each regional volume $V_r$ is replaced with its age-relative adjusted metric, denoted as $V'_r = V_r / V_s(a)$. Similarly, each regional surface area $S_r$ is transformed into an adjusted metric $S'_r = S_r / V_s(a)^{2/3}$^60^.

The feature vector corresponding to every subject contains the 20th, 40th, 60th, and 80th percentile of each morphological feature for every region. Morphological features don’t share the same units of measurement; for instance, the surface area is measured in $\textrm{mm}^2$, while volume is measured in $\textrm{mm}^3$. Consequently, we anticipate having different ranges of values, which might adversely affect the performance of the classifiers^61^.

We used min–max normalization between 0 and 1, which is one of the commonly used normalization methods in biomedical data.^62^ Thus, we normalized each column in the data matrix *D* using Equation (2).

2 $$\begin{aligned} \tilde{X}_{i,j} = \frac{X_{i,j} - \min _i\{{X_{i,j}}\}}{\max _i\{{X_{i,j}}\}-\min _i\{{X_{i,j}}\}} \end{aligned}$$

where $\tilde{X}_{i,j}$ denotes feature value *j* after normalization, and $X_{i,j}$ denotes the original value of feature *j*. *i* denotes the subject’s index within the data matrix, $\min {(X_j)}$ denotes the min value of feature *j*, and $\max {(X_j)}$ denotes max value of the feature *j*. $d_{tn}$ denotes the data matrix after the normalization for the behavioral groups where $1\le t\le 6$.

### Feature selection

To create a computer-aided diagnostic system (CAD) that accurately diagnoses autism, a neuro-atlas customized to the unique developmental patterns of the brain in autism must be employed. However, to the best of our knowledge, the existing literature on autism spectrum disorder lacks an ASD-specific neuro-atlas, or even a behavior-specific neuro-atlas that can be utilized to train machine learning (ML) classifiers and construct a CAD system. Therefore, in this study, we take the first steps toward building behavioral-based neuro-atlases that can be used to classify subjects into different ASD behavioral groups. In other words, we are proposing a neuro-atlas that can be used to objectively predict the severity score of a given subject if that subject was measured on one of the SRS scales. To achieve this goal, we utilize the recursive feature elimination with cross-validation (RFECV) method to select the set of brain morphological features which maximizes the balanced accuracy score of classifying ASD within each behavioral group. We divide the data within every behavioral group fivefold. The process for selecting the optimum number of features for classification involved conducting 5-fold cross-validation. First, a given classifier is trained on the training set, and we evaluate its performance using the validation set. The least significant feature is removed from the model, and the whole process was repeated until only one feature remains. The same process is repeated for each fold, we start, every time, by training the classifier using all the features, then using all features excluding the least significant one, and so on, until classification was done on a single feature. The average performance of the 5-fold cross-validation was calculated for each scenario, and the number of features for which the classifier had the maximum performance score was found, denoted $N_f$. In order to find the most significant $N_f$ features, we repeat the algorithm over all the subjects. For more information on the algorithm and its implementation, we refer the reader to Guyon et al.^63^ and Pedregosa et al.^64^, respectively.

For each of the behavioral groups, four different classifiers were used to implement RFECV and build a total of four models that aim to identify the set of features that maximizes the balanced accuracy score for classifying subjects into TD versus mild ASD, TD versus moderate ASD, and TD versus severe ASD. Therefore, for every behavioral group, there are 12 selected sets of features. Those 12 sets of features are divided into 4 sets of features that act as neuro-atlas candidates to describe and differentiate between TD and each severity level of ASD (mild, moderate, and severe). Each of the selected sets of brain morphological features maximizes the balanced accuracy score according to the underlying hypothesis of the utilized RFECV model. Each of the four selected models, to integrate with RFECV, possesses one of the following underlying hypotheses: (1) models that assume that classes are not linearly separable and thus select a set of features that constructs a feature space where the subjects are separated via non-linear kernel, and (2) models that assume that classes are linearly separable and thus search for a set of features that constructs a feature space, where the subjects are linearly separable. RFECV+RF, RFECV+Light Gradient Boosting Machines (LGBM) implement the first group of models with the underlying assumption that the classification accuracy can be maximized with a non-linear kernel in the selected feature space. On the other hand, RFECV+logistic regression (lr), RFECV+linear SVM (lsvm) implement the second group of models with the underlying that the classification accuracy can be maximized with a linear kernel in the selected feature space. The RFECV models are trained using 5-fold cross-validation. In each iteration, one of the 1088 features is removed, and a 5-fold cross-validation is performed on the remaining features to calculate the average balanced accuracy score.

This step outputs four sets of features for each severity level within each of the behavioral groups with a total number of 4 RFECV models $\times$ 3 severity levels $\times$ 6 behavioral groups = 72 sets of features. Those 72 sets of features are used to reduce the dimension of each of the data matrices of each behavioral group from 1088 columns/features to $s_{knm}$, where the size of $s_{knm}$ is $n \times m$ such that *n* is the number of subjects within behavioral group *k*, and $m \le 1088$. Consequently, we define a neuro-atlas for a specific behavioral group as the brain regions included with a selected set of morphological features which achieves the maximum value of the average of the balanced accuracy score and F1-score^65^ in the following ML step. More details are provided in the ML section. It is worth noting that the F1-score is only utilized at the ML step as a confirmation step of the ML models since all of the selected sets of features are chosen using solely the balanced accuracy score. This approach essentially means that the features selected serve as an educated estimate of what features we might exclude from the learning phase. Therefore, the feature selection part involves no hyperparameter optimization. Once the training phase concludes, we include only the selected features and the determined hyperparameter values. Subsequently, we evaluate the ’untrained’ classifiers on a hold-out set to verify the model’s generalizability.

### Scoring metrics

In this study, we used the balanced accuracy score as our first metric, which was introduced in 2010 to address the issue of optimistic estimates that can arise when a biased classifier is tested on an imbalanced dataset.^66^ Equation (3) defines the balanced accuracy score.

3 $$\begin{aligned} bacc= \frac{sensitivity+specificity}{2}, \end{aligned}$$

where *score* denotes the balanced accuracy score, *sensitivity* is calculated as $\frac{TP}{TP+FN}$ such that true positives are denoted by *TP*, false negatives are denoted by *FN*, and *specificity* is calculated as $\frac{TN}{TN+FP}$ such that *TN* denotes true negative, and *FP* denotes false positives.

The second metric that we utilized in this study is F-measure (F1), which is widely used in the context of the classification of imbalanced datasets.^67,68^ It was originally introduced to evaluate the ranking of documents retrieved based on a query.^69^ It is interpreted as the harmonic mean of the two degrees of freedom of a confusion matrix,

4 $$\begin{aligned} F1 = 2\times (\frac{PR}{P+R}), \end{aligned}$$

where *P* denotes precision which is calculated as $\frac{TP}{TP+FP}$, and *R* denotes recall which is calculated as $\frac{TP}{TP+FN}$. It is worth mentioning that we have not carried out any specific analysis to identify the optimal threshold for calculating sensitivity and specificity. Instead, we opted for a threshold of 0.5, which aligns with the default value employed in the scikit-learn package.

**Table 5:** Hyperparameter range of each classifier.

Classifier	hyperparameters	var_name	values
Linear SVM	Regularization parameter	C	0.1, 1, 5, 10
	Norm used in the penalization	penalty	$l_1$, $l_2$
	Loss function	loss	hinge, squared hinge
Ridge Classifier	Regularization parameter	$\alpha$	$\{0.1, 0.2, ldots, 5.0\}$
	Normalization	normalize	True, False
Logistic Regression	Norm used in the penalization	penalty	$l_1$, $l_2$, ElasticNet
	Regularization parameter	C	0.1, 1, 5, 10
	Algorithm to use in optimization problem	solver	Newton-CG, l-BFGS, liblinear, Sag, Saga
Light Gradient Boosting Machines	$l_1$ regularization	$\textrm{reg}_\alpha$	$\{0.0, 0.1, 0.2, \ldots , 5.0\}$
	$l_2$ regularization	$\textrm{reg}_\lambda$	$\{0.0, 0.1, 0.2, ldots, 5.0\}$
	Number of estimators	$n\_estimators$	$\{100, 150, \ldots , 5000\}$
	learning rate	$learning\_rate$	$\{0.01, 0.02, \ldots , 1.00\}$
	Maximum depth for each tree	$max\_depth$	$\{1, 2, \ldots , 10\}$
	Number of leaves	$num\_leaves$	$\{3, 4, \ldots , 32\}$
Random Forest	Number of trees in the forest	$n\_tree$	50, 100, 200, 500, 1000
	Function to measure the quality of a split	criterion	gini index, entropy
	Number of features to consider when looking for best split	$max\_features$	sqrt log2 All features
	Minimum number of samples required to split an internal node	$min\_samples\_split$	1, 2, 5, 10
	Whether to use bootstrap samples while building the tree or use the whole training set	bootstrap	True, False
Non-linear SVM	kernel used	kernel	polynomial, RBF, sigmoid
	Regularization parameter	C	0, 0.1, 1, 5, 10
	Degree (polynomial kernel only)	degree	2, 3, 4, 5, 6
	Kernel coefficient	gamma	scale, auto
	Independent term in kernel function	coef0	0.0, 0.01, 0.1, 0.5, 1, 5, 10, 50, 100

### ML classifiers

For every data matrix, after applying feature selection step, for every behavioral group *k*, an ML model is initialized to be trained to classify TD vs mild ASD, TD vs moderate ASD, and TD vs severe ASD. In this study, we decided to perform one vs one classification for multiple classes and to classify each of the ASD behavioral severity vs TD. The rationale behind this decision is that we are interested to see how the morphology of TD brain differs from the morphology of ASD with different severity scores without overanalyzing the discrepancies within the ASD itself. Throughout this study, we are focusing on our primary objective which is building ML-based neuro-atlases for ASD. ML classifiers, with a linear hypothesis and nonlinear hypothesis, are selected to classify subjects within every behavioral group. The training process is repeated three times for classifying subjects into TD and each of the severity levels, similar to the experiment design of feature selection. The six ML classifiers used in this study are categorized into two main groups: (1) Linear classifiers, which include LR, LSVM, and Ridge classifier; and (2) Non-linear classifiers, which include RF, SVM with radial basis function (SVM-RBF), and LGBM. The reduced data matrix $s_{kn}$ of each behavioral group *k* is fed to every classifier for training. For each classifier, the hyperparameters and their ranges are defined in Table 5. The random sampling of the hyperparameter space is repeated 500 times for each classifier. The hyperparameters of each classifier corresponding to the maximum average cross-validation balanced accuracy score are selected as the output of this stage.

There is an extra step is added to the end of the ML pipeline in order to make sure that the selected ML model is not overfitted. Each of the optimized ML classifiers is re-initialized with the optimum hyper-parameter’s values, retrained using only 80% of the data, and tested on the 20% left out. The testing balanced accuracy and F1 score are calculated for each of the retrained classifiers to compare against the cross-validation score.

### Building neuroatlases

The complexity of the behavioral classification step makes it vulnerable to false positive findings because of one or more potential hidden confounding variables that might exist among a group of subjects in the dataset. In general, a complex experiment, especially in biomarker studies, possesses many statistical caveats^70^. Moreover, the behavioral classification step comprises two stochastic processes that would change the final results for different initial conditions. The two stochastic processes are: (1) shuffling subjects for cross-validation, and (2) hyperparameter optimization via random search. Although we sampled the hyperparameter space of every ML classifier 500 times, there is no guarantee that the resultant model is globally optimal, at least for the data in hand.

We executed the behavioral classification step 51 times with different random seeds for each of the two stochastic processes. The rationale behind repeating the behavioral classification step is to be more confident with both the selected morphological features, and the optimized model. We claim that robust discriminative morphological features, which can be generalized outside ABIDE II dataset, should be consistently selected by RFECV across different samples of training-validation sets. Consequently, that robust discriminative set of morphological features should yield the highest possible accuracy scores when utilized with a hyperparameter-optimized ML classifier.

Moreover, we were intrigued by the question of whether the set of morphological features, that corresponds to the maximum classification accuracy for each behavioral group, are selected due to chance, or they actually reflect some sort of underlying neurophysiology that defines ASD. Therefore, we calculated the probability of finding each of the selected morphological features for *n* number of times out of the 51 trials using binomial distribution as going to be explained in the following section.

### Neuro-atlas statistical significance

Testing the significance, of each of the selected features within each neuro-atlas, was a challenging task, especially given the way we designed the framework. The framework is primarily designed to build a CAD system and annotate the features that maximize the classification accuracy of that CAD system. However, studying the significance of the findings is as important as the classification accuracy, if it is not more important. Therefore, we set the null hypothesis, and then analytically calculated the probability of including a specific feature in a specific neuro-atlas under the given null hypothesis.

The null hypothesis is that RFECV algorithms randomly select *m* features of each behavioral group data matrix. Thus, over the 51 repetitions, what is the probability of observing the neuro-atlas features under the assumption of the null hypothesis? To answer this question, consider the RFECV algorithm running in reverse, where it selects a feature, instead of eliminating a feature, up to *m* features. Therefore, for every repetition, the probability of randomly selecting any feature out of 1088, through *m* samplings without replacement, is *m*/1088 such that *m* is the number that RFECV selected for this behavioral group for this severity level for this repetition out of the 51 repetitions. Let feature $f_i$ such that $1\le i \le 1088$ gets selected *r* times out of the 51 repetitions, then the probability that $f_{i}$ is randomly selected *r* times is defined as

5 $$\begin{aligned} P(f_i; 51, r) = \left( {\begin{array}{c}51\\ r\end{array}}\right) \left( \frac{m}{1088}\right) ^{r} \left( 1-\frac{m}{1088}\right) ^{51-r} \end{aligned}$$

Equation (5) is the well-known binomial distribution equation^71^. Although equation (5) is too close to what we are looking for, it is not exactly the correct representation of our experiment. As we described the stochastic part of the behavioral classification step in the last section, we can not ensure that for the same behavioral group, for the same severity level, the same *m* will be selected for every repetition. Therefore, a generalization of equation (5) is required to be applied to our case. We can reformulate our question to be: What is the probability of including feature $f_i$ *r* times in the selected feature set in a total of 51 experiments given that the probability of selecting $f_i$ varies across experiments? After introducing the variability of the probability of success of each feature based on the experiment, we can no longer calculate the probability $P(f_i; 51, r)$ using $$\left( {\begin{array}{c}51\\ r\end{array}}\right)$$. Therefore, we implemented a function that counts all the ways such that $f_i$ can be observed *r* times giving the probability of selecting $f_i$ at every experiment. The code can be found at^72^ in the subdirectory *notebooks*/*probabilityOfSuccessComputations*.*py*. The statistical significance of the selected feature is performed on the neuro-atlas of every behavioral group, and on the aggregated neuro-atlases to inspect the most common features among all ASD-related behavioral disorders.

Although the significance would be calculated exactly using the previous technique, the number of combinations we need to consider grows exponentially and becomes impossible to track. For instance, the number of combinations required to be considered to calculate the significance of selecting one feature 10 times out of the 51 experiments is $$\left( {\begin{array}{c}51\\ 10\end{array}}\right) = 12,777,711,870$$. Therefore, for every feature, we assigned the highest selection probability that features possessed across the 51 experiments as the *p* defined in the binomial distribution. Afterward, we used the binomial distribution equation (5). This crude assumption might result in type II error which would lead to ignoring significant brain regions. Nevertheless, in this work, we attempted to be as conservative as possible while building the neuroatlases. Consequently, those brain regions, which are found to be significant, can be thought of as the “core” brain regions that are directly associated with the disorder. A brain region is said to be significantly associated with ASD within a severity group of a behavioral group if and only if at least one of the morphological features is utilized in one of the selected ML models of the 51 experiments, and has a p-value< 0.001.

Eventually, since the behavioral classification step is a supervised process, we wanted to study the relationship between the top-performing models across all experiments testing and the behavioral groups as well as the relationship between the top-performing models and the severity groups. We define the top-performing models as the top 50th percentile models in terms of average cross-validation balanced accuracy score, testing balanced accuracy score, and testing F1-score. The rationale behind studying the aforementioned relationships is to provide complementary information to the statistical significance of every neuro-atlas. Substantially, with every neuro-atlas, we provide information on how consistent and accurate a machine learning model would be if it is trained using a given neuro-atlas.

## Conclusion and future work

In this study, we presented a pioneering method for the diagnosis of autism and other psychological disorders through the replication of the clinical diagnosis process using artificial intelligence. Our proposed framework consists of two crucial stages in diagnosing a subject with Autism Spectrum Disorder (ASD). Firstly, we obtain morphological features from the MRI scans of each subject and identify the most salient features that accurately differentiate ASD within various behavioral domains. Secondly, we categorize each subject as having severe, moderate, mild, or typical development (TD) based on the behavioral domains of the SRS. The developed model demonstrated an impressive average accuracy of 0.96. This accuracy was calculated as the mean across the top-performing AI models for both feature selection and severity classification within various behavioral groups. It is also important to note that the current diagnostic process for ASD relies heavily on reports based on patient interviews and physician-based scores, which can be time-consuming and susceptible to human error. The ability of our AI-based model to detect functional differences in brain regions using MRI scans alone can not only speed up the diagnostic process but also increase its accuracy, leading to improved outcomes for individuals with ASD. The proposed framework also provides clarification and interpretation of the classifier’s decisions at every step. During the training of the classifiers, we constructed neuroatlases to gain insight into the correlation between brain region morphology and various behavioral traits of each subject. The regions of the brain defined in each behavioral neuroatlas were chosen based on a combination of machine learning classification efficiency and statistical significance. Finally, interpretable methods were employed to demonstrate, for new subjects, the mechanisms and reasons behind their classification/diagnosis. This interpretability phase has been included to assist physicians in comprehending the fundamental causes of ASD and to enable them to offer personalized medical treatment for each subject.

In the future, this research can expand its impact by increasing the dataset’s size and diversity, using different types of brain data, and tracking the progression of ASD over time. It is also important to note that the field of neuroscience is always evolving and the understanding of the function of different brain regions is still under research.

## Data Availability

This work uses rs-fMRI data from the ABIDE-II dataset, publicly available at ABIDE II website. The website also contains demographic information and scanning parameters that were used.

## Code availability

Codes are available on the following github repository Behavioral Classification codes.
